# Identification of AS1842856 as a novel small‐molecule GSK3α/β inhibitor against Tauopathy by accelerating GSK3α/β exocytosis

**DOI:** 10.1111/acel.14336

**Published:** 2024-09-17

**Authors:** Da‐Long He, Xiao‐Yu Zhang, Jing‐Yang Su, Qi Zhang, Ling‐Xiao Zhao, Ting‐Yao Wu, Hang Ren, Rong‐Jun Jia, Xian‐Fang Lei, Wen‐Jia Hou, Wen‐Ge Sun, Yong‐Gang Fan, Zhan‐You Wang

**Affiliations:** ^1^ Key Laboratory of Medical Cell Biology of Ministry of Education, Key Laboratory of Major Chronic Diseases of Nervous System of Liaoning Province Health Sciences Institute of China Medical University Shenyang China; ^2^ CAS Key Laboratory of Separation Science for Analytical Chemistry, Dalian Institute of Chemical Physics Chinese Academy of Sciences Dalian China; ^3^ First Affiliated Hospital of Jinzhou Medical University Jinzhou China; ^4^ Department of Radiology The First Hospital of China Medical University Shenyang China

**Keywords:** AS1842856, exocytosis, glycogen synthase kinase‐3α/β, neurodegenerative diseases, Tau hyperphosphorylation

## Abstract

Glycogen synthase kinase‐3α/β (GSK3α/β) is a critical kinase for Tau hyperphosphorylation which contributes to neurodegeneration. Despite the termination of clinical trials for GSK3α/β inhibitors in Alzheimer's disease (AD) treatment, there is a pressing need for novel therapeutic strategies targeting GSK3α/β. Here, we identified the compound AS1842856 (AS), a specific forkhead box protein O1 (FOXO1) inhibitor, reduced intracellular GSK3α/β content in a FOXO1‐independent manner. Specifically, AS directly bound to GSK3α/β, promoting its translocation to the multivesicular bodies (MVBs) and accelerating exocytosis, ultimately decreasing intracellular GSK3α/β content. Expectedly, AS treatment effectively suppressed Tau hyperphosphorylation in cells exposed to okadaic acid or expressing the Tau^P301S^ mutant. Furthermore, AS was visualized to penetrate the blood–brain barrier (BBB) using an imaging mass microscope. Long‐term treatment of AS enhanced cognitive function in P301S transgenic mice by mitigating Tau hyperphosphorylation through downregulation of GSK3α/β expression in the brain. Altogether, AS represents a novel small‐molecule GSK3α/β inhibitor that facilitates GSK3α/β exocytosis, holding promise as a therapeutic agent for GSK3α/β hyperactivation‐associated disorders.

## Graphical abstract

AS1842856 (AS) inhibits Tauopathy via promoting glycogen synthase kinase‐3α/β (GSK3α/β) exocytosis. Upon entering the cell, AS binds to GSK3α/β and facilitates the translocation of GSK3α/β to early endosomes, which in turn promotes GSK3α/β accumulation in multivesicular bodies (MVBs) and subsequently reduces intracellular GSK3α/β contents via promoting GSK3α/β exocytosis. Reduced intracellular GSK3α/β inhibits Tau hyperphosphorylation and the formation of neurofibrillary tangles.

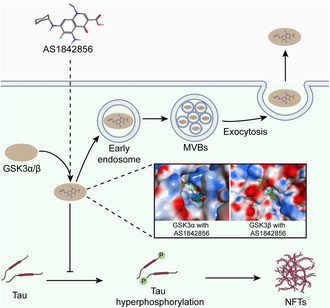

**Abbreviations**
AD: Alzheimer's disease
Alix: apoptosis‐linked gene 2‐interacting protein 1
AS: AS1842856
ATP1A1: ATPase Na+/K+ transporting alpha 1
BLI: Bio‐layer interferometry
CETSA: cellular thermal shift assay
FOXO1: forkhead box protein O1
GSK3α/β: glycogen synthase kinase‐3α/β
IL: interleukin
MAP 2: microtubule‐associated protein 2
MVBs: multivesicular bodies
NFTs: neurofibrillary tangles
NMDA: N‐methyl‐D‐aspartate
PP2A: protein phosphatase 2A
PSD95: postsynaptic density protein 95
SYP: synaptophysin
TNF‐α: tumor necrosis factor‐α

## 1 INTRODUCTION

Tau hyperphosphorylation is an outstanding pathological characteristic of neurodegenerative diseases such as Alzheimer's disease (AD), frontotemporal dementia (FTD), and progressive supranuclear palsy (PSP). It leads to the formation of insoluble aggregates, microtubule instability, altered cytoskeletal stability, and loss of axonal transport integrity (Sayas & Ávila, 2021), resulting in the formation of neurofibrillary tangles (NFTs) and impaired neurological transmission (Noble et al., 2013). Currently, the anti‐Tau immunotherapies were suggested as potential interventions in AD, while most of the antibodies targeting Tau proteins failed in the clinical trials (Congdon et al., 2023; Monteiro et al., 2023), suggesting that the other strategies should also be considered for the treatment of Tau pathology.

Tau hyperphosphorylation is modulated by multiple enzymes, such as glycogen synthase kinase‐3 (GSK3), cyclin‐dependent kinase 5 (CDK5), and protein phosphatase 2A (PP2A), and so forth (Iqbal & Grundke‐Iqbal, 2008). GSK3, comprising two subtypes, GSK3α and GSK3β, plays a crucial role as a key kinase in the hyperphosphorylation of Tau (Zhao et al., 2024). Mounting evidence indicates that when GSK3α/β is hyperactivated, it promotes Tau hyperphosphorylation and disrupts several neurometabolic activities during the development of AD (Chauhan et al., 2022; Wu et al., 2022). Inversely, inhibition of GSK3α/β dramatically reduces Tau hyperphosphorylation and aggregation, as well as improves the cognitive capacity in AD animal models (Amaral et al., 2021; Liu et al., 2024). Of note, overexpression and overactivation of GSK3α/β were observed in the brains of AD patients (Pláteník et al., 2014; Zhou et al., 2022), suggesting that targeting GSK3α/β is an attractive strategy for treating AD. Regrettably, the regulatory network governing GSK3α/β remains incompletely understood, and the intricate dynamics of intracellular GSK3α/β are yet to be fully unraveled. This significant gap in knowledge poses a substantial barrier to advancing targeted therapies aimed at GSK3α/β. Furthermore, although several GSK3α/β inhibitors were developed (Fronza et al., 2023; Li et al., 2023), the applications of these inhibitors for AD therapy remain unreachable because of their large molecular weight, serious side effects, low efficacy, among others (Wei et al., 2022). Therefore, developing novel small molecular inhibitors of GSK3α/β may shed new light on the treatment of Tau hyperphosphorylation‐related diseases.

AS1842856 (AS) was used as a small molecular inhibitor of forkhead box O1 (FOXO1) (Nagashima et al., 2010). It was suggested to decrease blood glucose and attenuate diabetic complications in mice modeled with type II diabetes (Shi et al., 2018). Moreover, AS also has the pharmacological functions to improve cardiac functions and ameliorate acute lung injuries (Artham et al., 2019; Spurthi et al., 2018). Additionally, AS exerted good biosafety as evidenced by a long‐term treatment of AS in mice does not lead to any significant side effects (Liu et al., 2019). Considering the progression of AD is highly associated with diabetes and cardiac functions (Hendriks et al., 2023; Oh et al., 2023), the beneficial effects of AS in diabetes and cardiac functions render us to further explore the pharmacological functions of AS in Tau pathology. Interestingly, during our performance of the non‐targeted prediction database (www.swisstargetprediction.ch), we discovered that GSK3β is the most probable potential target of AS (Table S1). Therefore, we asked whether AS can influence the expression or activity of GSK3, and in turn, regulate Tau phosphorylation.

In this study, we first demonstrated that AS could directly bind to GSK3α/β, and subsequently reduce intracellular GSK3α/β levels via accelerating GSK3α/β exocytosis. Furthermore, AS could effectively penetrate the blood–brain barrier (BBB) and long‐term treatment of AS reduced GSK3α/β expression and inhibited Tau hyperphosphorylation in the brains of P301S transgenic mice. Thus, accelerating GSK3α/β exocytosis is a potential strategy to inhibit Tau pathology, and AS may be a potential drug for treating Tau hyperphosphorylation‐related neurodegenerative disorders.

## 2 MATERIALS AND METHODS

### 2.1 Molecular docking

The 3D structure of GSK3α was downloaded from AlphaFold with the ID: Q2NL51, and the 3D structure of GSK3β was downloaded from Protein Data Bank (PDB) with the PDB ID: 6AE3. The 3D structures of GSK3α and GSK3β were edited with “QuikPrep,” and the 3D structure of AS1842856 was for energy minimization. The Placement method of dock with Triangle Matcher and Refinement method is Rigid Receptor. The result of Ligand interactions was exported by “computer”‐“Ligand interactions” using MOE software.

### 2.2 Cell culture and treatments

N2a cells, LN229 cells, HEK293T cells, BV2 cells, HepG2 cells, and MN9D cells were cultured in high glucose dulbecco's modified eagle medium (DMEM) supplemented with 10% fetal bovine serum (FBS) and 1% penicillin/streptomycin (PS) in a constant temperature 37°C dressing box with 5% CO_2_. SH‐SY5Y cells were cultured in DMEM/F12 supplemented with 15% FBS and 1% PS. Every 2 days, the fresh medium was replaced for cells, and the culture density was adjusted to 80% for passaging. All cells were serum starved with FBS‐free medium for 4 h before compound treatment. The information on compounds used in this study is shown in Table S3. N2a cells were pretreated with MG132, CQ, CCCP, PMSF, Dynasore, Vacuolin‐1, Marimastat, Endosidin 2, and GW4869 for 2 h, and subsequently treated with AS for 6 h. For SH‐SY5Y cells, AS was first added to culture for 3 h, and then, the OA was added to co‐culture for 3 h. The vehicle group was added to the solvent control at the same dose.

### 2.3 Animal treatment

The P301S transgenic mice [B6C3‐Tg (Prnp‐MAPT*P301S) PS19 Vle/J] were originally obtained from the Jackson Laboratory (Bar Harbor, ME, USA). All animals were maintained at room temperature in an SPF barrier environment with 12 h alternating light/darkness and free available food and water. In this study, a total of 12 4‐month‐old male P301S mice were randomly divided into two equal groups. AS was dissolved in physiological saline containing 1% dimethyl sulfoxide (DMSO) and subsequently sonicated to promote dissolution. One group of mice was intraperitoneally injected with the AS (5 mg/kg) daily for 8 weeks. Another group of mice was injected with the same volume of solvent control at the same times. After that, mice were submitted to the behavior tests.

This study was carried out according to the recommendations of “Laboratory Animals‐Guideline of Welfare and Ethics, The Ethics Committee for Medical Laboratory Animals of China Medical University” (reference number: CMU2023517). The protocol was approved by The Ethics Committee for Medical Laboratory Animals of China Medical University.

### 2.4 Tissue preparation

Twenty‐four hours after the last intraperitoneally injection with either AS or vehicle, mice were anesthetized using sodium pentobarbital (50 mg/kg, intraperitoneally). Subsequently, mice were perfused with physiological saline via transcardium and sacrificed by decapitation. The brains were promptly removed and dissected into halves on a chilled board. One half was fixed in 4% polyformaldehyde for morphological assessment, while the other half was frozen at −80°C for biochemical analyses.

### 2.5 Bio‐layer interferometry (BLI) assay

The recombinant proteins of GSK3α and GSK3β with His tag were purchased from CLOUD‐CLONE (Wuhan, China) (Cat: RPA360Hu01) and Sino Biological (Cat: 10044‐H07B). The binding assay of the AS to GSK3α and GSK3β was performed using the Octet K2 system. GSK3α and GSK3β were loaded onto the Ni‐NTA biosensor, respectively. Then, different concentrations of AS solutions were prepared using PBS (1.06, 3.13, 6.25, 12.5, 25, 50, 100 μM). Then, the biosensors loaded with GSK3α and GSK3β were exposed to different concentrations of AS solution. The data were analyzed by Forte Bio (Date Analysis 11.0).

### 2.6 Cellular thermal shift assay (CETSA)

The CETSA was performed according to a previous report with some modifications (Martinez Molina et al., 2013). Briefly, N2a cells were cultured at 80% density in DMEM with 10% FBS, the medium was removed by PBS, and cells were digested with 0.25% trypsin. The supernatant was discarded after 3 min at 1000 rpm, and the cells were resuspended with 1 mL PBS. Cells were snap‐frozen in liquid nitrogen, thawed in room temperature water, and repeated three times. The lysate was centrifugated at 10,000 rpm for 20 min at 4°C, and the supernatant was aspirated and divided equally into two 1.5 mL EP tubes. 5 μL of AS (500 μM) was added into one tube, and another tube was added with solvent control. After incubation for 30 min at room temperature in darkness, the mixtures were equally divided into the 200 μL EP tubes. The tubes were heated for 3 min under a temperature gradient (43.9°C, 46.8°C, 50.5°C, 54.8°C, 59.4°C, 64.2°C) and left for 5 min at room temperature. The mixtures were added with protein loading buffer for western blot detection.

### 2.7 Cell viability assay

Cell viability was determined using the Cell Counting Kit‐8 (CCK8) method. SH‐SY5Y cells were seeded in 96‐well plates until 60% confluence. After different concentrations of AS (0, 0.25, 0.5, 1, 2, 4, 8 μM) treatment for 24 h, cells were added with CCK8 reaction solution (MCE, HY‐K0301) and further incubated at 37°C for 2 h. The absorbance of each well was recorded using a microplate reader (Thermo Fisher Scientific, 1510) at a wavelength of 450 nm. The percentage of cell viability relative to the vehicle group was calculated. Experiments were repeated at least three times in quadruplicate.

### 2.8 Enrichments and detections of proteins in the medium

We used two different methods to enrich the proteins in the cell culture medium. In the experiment of pre‐chilled acetone enrichment of medium proteins, an equal number of N2a cells were seeded into four 6 cm dishes. When the number of cells grew to 80% of the culture area, cells were further incubated with a serum‐free medium, and the cells and mediums were collected after 12 h. Before the collection, cells were treated with AS (0.5 μM) for 3, 6, and 12 h. The collected mediums were added to 3 times the volume of the pre‐chilled acetone, mixed and left to stand for 15 min at −20°C. The mediums were centrifuged at 3000 rpm for 20 min at 4°C. The precipitates were lysed and subsequently assayed using western blot. The expression of β‐actin in cells was used as the internal control.

In the method of immunomagnetic bead enrichment, an equal number of N2a cells were seeded into three 6 cm dishes. These were divided into vehicle, control, and AS groups. When the number of cells grew to 80% of the culture area, the serum‐free mediums containing AS (0.5 μM) were used to culture for 12 h in AS groups, and the vehicle and control groups were cultured for 12 h in serum‐free mediums. Cells and mediums were collected. Each of the mediums was added with 20 μL of magnetic beads (Invitrogen, 1003D), and mediums from control and AS groups were added with 4 μL of mouse anti‐GSK3α/β antibody (Affinity, BF8003), and medium from vehicle‐treated cells was added with 4 μL of mouse IgG antibody (Cell Signaling Technology, 5873S). The mediums were spun overnight at 4°C. The beads were washed and added with 50 μL protein loading buffer, the mixture was boiled for 5 min and sedimented, and the supernatant was used for western blot analysis. To address the disturbance of the protein‐heavy chain, rabbit anti‐GSK3α/β antibody (Immunoway Biotechnology Company, YT2081) was used in western blot detection. Meanwhile, the expression of β‐actin in cells was used as the internal control.

### 2.9 Transfections

Cells were seeded into 6‐well plates and further cultured to a density of 30%–40%. Cells were replaced with the serum‐free medium and transfected with 100 pM of siRNA or 2 μg plasmids per well using Trans‐geneverTM transfection reagent (GENEVER, B07001) according to the manufacturer's instructions. The siRNAs used as follows: human FOXO1 siRNA: 5′‐UUAUCUCAGACAGACUGGGTT‐3′; mouse FOXO1 siRNA: 5′‐GCAGCAGACACCAUGCUAUTT‐3′; mouse Rab5 siRNA: 5′‐GCAACAAGACCCAACGGGCCAAATA‐3′. The plasmids used as follows: pLV2‐CMV‐MAPT (human)‐P301S‐3×FLAG (Miao Ling Plasmid); GSK3β cDNA ORF Clone, Human, N‐GFPSpark® tag (Sino Biological, HG10044‐ANG). Cells were further cultured for 48 h before being treated with compounds.

### 2.10 Immunofluorescence

Mouse brains were cut on a cryostat (Leica, SM2010R) at a thickness of 30 μm. A series of three equally spaced brain sections (~1 mm apart) were used for each type of stain. The slides or cells were fixed with 4% paraformaldehyde for 10 min and subsequently permeabilized with 0.2% Triton X‐100 for 5 min at room temperature. After blockage with 5% Bull Serum Albumin (BSA; Sigma‐Aldrich, V900933) for 1 h, sections or cells were incubated with primary antibodies overnight at 4°C. The primary antibodies used in this study are shown in Table S2. The secondary antibodies, goat anti‐mouse‐Ig G Alexa 488 (Thermo Fisher Scientific, A‐11001, 1:300), goat anti‐rabbit‐Ig G Alexa 488 (Thermo Fisher Scientific, A‐11008, 1:300), goat anti‐mouse‐Ig G Alexa 594 (Thermo Fisher Scientific, A‐11005, 1:300), goat anti‐rabbit‐Ig G Alexa 594 (Thermo Fisher Scientific, A‐11037, 1:300), and DAPI (Solarbio, C0060, 1:200) were stained for 1 h at room temperature. Images were obtained using a confocal laser microscope (Nikon A1), and the fluorescent intensities were quantified using Image J software.

### 2.11 Real‐time polymerase chain reaction

Total RNA from N2a cells was prepared using the RNA Extract kit (Accurate Biology, AG21101) and reverse transcription of RNA to cDNA using Fast King cDNA First Strand Synthesis Kit (Tiangen Biotech, China). Quantitative PCR (qPCR) was performed using a Real‐time quantitative PCR kit (Accurate Biology, AG11701) and a Light Cycler 96 system (Roche, Switzerland). Melting curve analysis was performed to confirm the production of a single product in each reaction. The mRNA expression was calculated using ΔΔCt (threshold cycle, Ct) values normalized to β‐actin. Sequences of primers are as follows: GSK3α: forward, 5′‐GGAGTATGTGCCCGAGAC‐3′ and reverse, 5′‐CACCTTGGGAGTGGATGT‐3′; GSK3β: forward, 5′‐CCACCATCCTTATCCCT‐3′ and reverse, 5′‐CAGAAGCGGCGTTATTG‐3′; β‐actin: forward, 5′‐CATCCGTAAAGACCTCTATGCCAAC‐3′ and reverse 5′‐ATGGAGCCACCGATCCACA‐3′.

### 2.12 Live cell imaging

HEK293T cells were inoculated into 24‐well plates and transfected using GSK3B cDNA ORF Clone, Human, N‐GFPSpark® tag purchased from Sino Biological (HG10044‐ANG). After that, Dil (Solarbio, G1705‐1, 1:1000) was used to label cell membranes as red. Cells were first pretreated with AS (0.5 μM) or vehicle for 1 h. Then, five fields of view were monitored in each well, and pictures were captured every 2.5 min, for a total of 3 h of image acquisition. During this experiment, cells were kept at a constant temperature of 37°C with 5% CO_2_, and imaging was carried out using Nikon Biopeline.

### 2.13 Cycloheximide (CHX) chase experiments

CHX was purchased from Caymanchem (14126). A total of 5 × 10^5^ N2a cells were seeded onto six‐well plates to culture for 24 h and subsequently starved in FBS‐free medium for 4 h. Cells were treated with 0.5 μM AS or/and CHX (80 μM) for various periods (0, 3, 6, 9, and 12 h). Then, the cells were harvested and analyzed by western blot.

### 2.14 Imaging mass spectrum analysis

For sample preparation for imaging mass microscope (iMScope TRIO), the method was developed based on our previous study (Zhang et al., 2022). In brief, the frozen brain tissues were cut at 10 μm and mounted on an electrically conductive glass slide‐bearing specimen. Subsequently, a “two‐step matrix application,” which combined with sublimation and airbrushing, was used to coat matrix α‐cyano‐4‐hydroxycinnamic acid (CHCA) for tissue sections. iMScope analysis was performed using a 1000 Hz solid laser. A 45‐μm pitch of special resolution was used, and the data were acquired in positive ionization. The m/z values were internally calibrated with 2,5‐dihydroxybenzoic acid (DHB). All the spectra were acquired using atmospheric pressure matrix‐assisted laser desorption/ionization (MALDI) (Shimadzu Corporation). The laser in the iMScope system was a diode‐pumped 355 nm Nd: YAG laser (Shimadzu Corporation, Kyoto, Japan) and operated under the following parameters: frequency, 1000 Hz; laser intensity, 45; laser diameter, 2. The parameters of IT‐TOF MS were set as follows: ion polarity, positive; mass range, 200–400; sample voltage, 3.5 kV; detector voltage, 1.70 kV. The imaging MS Solution Version 1.30 software (Shimadzu, Tokyo, Japan) was used to control the instrument, and the data acquisition, visualization, and quantification were also performed using the same software. The identities of AS were further confirmed by MALDI‐MS/MS with reference to authentic standard (HY‐100596, MCE).

### 2.15 Vesicle enrichment in brain lysates

The experimental procedure for enriching vesicles in brain lysates was appropriately modified, considering previously reported protocols (Tanaka et al., 2016). Briefly, an appropriate 3 uL per sample amount of mouse anti‐apoptosis‐linked gene 2‐interacting protein 1 (Alix) (Cell Signaling Technology, 2171) was mixed with pre‐cooled magnetic beads on ice and further incubated for 1 h. 1 mL of 10 mM N‐2‐hydroxyethylpiperazine‐N‐ethane‐sulphonicacid (HEPES)‐sucrose buffer (10 mM HEPES [pH 7.4], 320 mM sucrose, 5 mM MgSO_4_, 1 mM Ethylene Diamine Tetraacetie Acid (EDTA), and protease inhibitors) homogenized an amount of 50 mg cerebral cortex. In total, 80 μL of each sample was mixed for IgG groups, and 100 μL of each sample was transferred for input. The mixtures were then spun overnight with the antibody‐coupled magnetic beads at 4°C, and the IgG group mixtures were spun overnight with an equal amount of magnetic beads conjugated mouse IgG antibody (Cell Signaling Technology, 5873S). The samples were washed 5 times with 1 mL of HEPES‐sucrose buffer on a magnetic rack. Finally, samples were eluted with sample buffer and heat denatured at 100°C for 5 min for subsequent western blot detection.

### 2.16 Western blot

Cells or tissues were lysed by RIPA (Beyotime Biotechnology, P0013C) supplemented with 1% phenylmethylsulfonyl fluoride (PMSF, MCE, HY‐B0496), 1% protease inhibitor (MCE, HY‐K0010), and 1% phosphatase inhibitor (MCE, HY‐K0022). The lysates were centrifuged at 10,000 rpm for 20 min at 4°C. The supernatants were collected, and the protein concentrations were determined using the BCA kit (Beyotime Biotechnology, P0009) according to the manufacturer's instructions. A total of 20–40 μg proteins were loaded into SDS‐PAGE polyacrylamide gels; then, proteins were transferred onto PVDF membranes (Millipore, IPVH00010). PVDF membranes were blocked in 5% nonfat milk for 1 h at room temperature and incubated with primary antibody overnight at 4°C. The primary antibodies used in this study are shown in Table S2. Membranes were washed with TBST and subsequently incubated with horseradish peroxidase (HRP)‐labeled secondary antibodies for 1 h at room temperature. Enhanced chemiluminescence (ECL) kits (Tanon, 180‐5001) and Tanon 5200 with Tannon Allcap software were employed to detect blots. Data from the bands were determined using Image J software.

### 2.17 Morris water maze (MWM) test

The MWM test was applied in a circular pool of 1.5 m diameter, with a depth of 40 cm and a moderate amount of milk to make the water opaque. In the southwest (SW) quadrant of the pool, a circular platform with a diameter of 10 cm was placed. The different shapes of signs are pasted on the walls of the pool in the four quadrants. The platform period is 7 days in total, divided into visible platform tests and hidden platform tests, where the visible platform tests were the first 2 days, and the hidden platform tests were the last 5 days. A limit of 1 min was given for the mice to find the visible platform and hidden platform. In visible platform tests, the platform was 1 cm above the water surface, and the mice entered the water from three different quadrants daily. If the mice could not find the platform within 60 s, the mice were guided to the platform and stayed on the platform for 30 s. In hidden platform tests, the water surface is 1 cm above the platform and the rest of the process is the same as visible platform tests. On the eighth day of the experiment, the platform was removed, and the number of times that the mice crossed the platform region was recorded for 1 min. All the data were recorded and analyzed with a computer program (Panlab, Smart 3.0).

### 2.18 Statistical analysis

All the experiments and analyses are conducted with the experimenter blind to drug treatment. The Student's *t*‐test was performed to calculate the level of significance for comparing two groups, while one‐way analysis of variance (ANOVA) or two‐way ANOVA followed by the Sidak *post hoc* test was done for comparing more than two groups. All datasets were tested for normal distribution and homogeneity of variance to confirm that non‐parametric testing was not required. In multigroup studies with parametric variables, *post hoc* tests were conducted only if F in ANOVA (or equivalent) achieved the “chosen” necessary level of statistical significance and there was no significant variance in homogeneity. The sample of all animals was 6 and co‐localization analyses were performed on three sections per sample and averaged, and three technical replicates were used for all cellular experiments. All graphs and statistics were done in GraphPad Prism 8. All data sets were tested for outliers using the Grubbs test. All data were expressed as mean ± SEM, and the significance level for all analyses was set at *p* < 0.05.

## 3 RESULTS

### 3.1 AS treatment reduces GSK3α/β levels through a non‐transcriptional pathway

**FIGURE 1:**
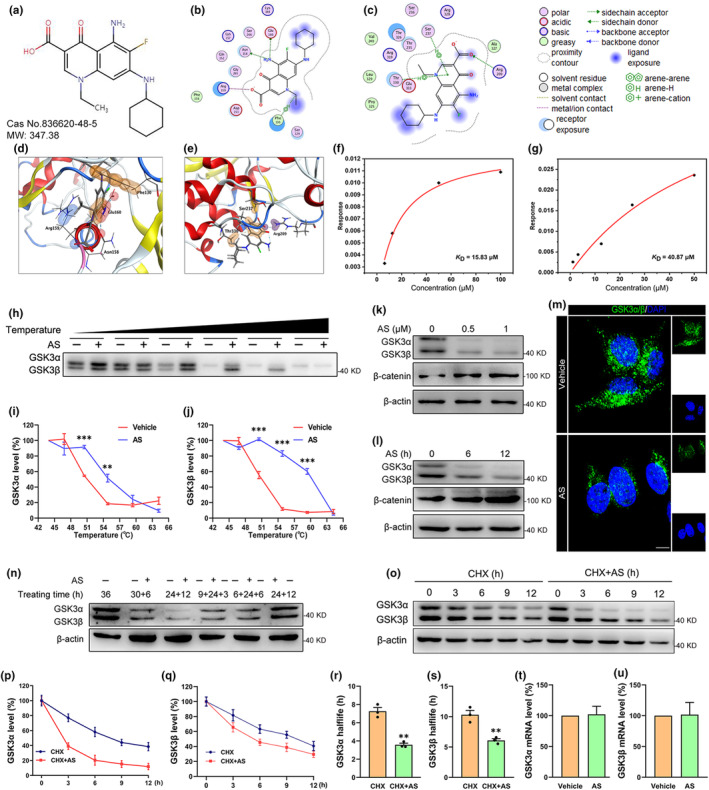
AS treatment reduces GSK3α/β levels through a non‐transcriptional pathway. (a) The molecular structure of AS. (b–e) Ligand interaction images between AS and GSK3α (b) or GSK3β (c). Schematic view of AS binding to GSK3α (d) or GSK3β (e). (f, g) Binding affinity of AS to GSK3α (f) and GSK3β (g), the K_D_ values are shown. (h–j) Western blot quantified the level of GSK3α/β after AS treatment in CETSA (h); the melting curves of GSK3α (i) and GSK3β (j) were affected by temperature. (k) Western blot depicting changes in the levels of GSK3α/β and β‐catenin in N2a cells following AS treatment for 12 h. (l) Western blot depicting changes in the levels of GSK3α/β and β‐catenin in N2a cells following 0.5 μM AS treatment for 6 and 12 h. (m) The representative images showed that the fluorescence intensity of GSK3α/β (green) decreased in N2a cells after treatment with 0.5 μM AS for 6 h. The nuclei were marked by DAPI (blue). Scale bar, 10 μm. (n) Withdrawing AS rescued AS‐induced GSK3α/β reduction in N2a cells. (o–s) CHX (80 μM) plus AS (0.5 μM) treatment accelerated the reduction in intracellular GSK3α/β and decreased the halflife of GSK3α and GSK3β in N2a cells. (t, u) AS treatment did not change the mRNA expressions of both the GSK3α and GSK3β in N2a cells. *n* = 3; **p < 0.01, ***p < 0.001.

Since the GSK3β is the most probable target of AS (Table S1), we performed the molecular docking studies. As shown in Figure 1b–e, AS was visualized to bind to the GSK3α at Arg159, Asn158, Glu160, and Phe130, and GSK3β at Ser237, Thr330, and Arg209 using Molecular Operating Environment (MOE) software. Additionally, these amino acids are highly conserved in different species (Figure S1a,b), suggesting that AS could also bind to GSK3α and GSK3β in other species. BLI assay is widely used for detecting the interaction between proteins and small molecular compounds. Our study revealed that AS effectively bound to the GSK3α and GSK3β with K_D_ values of 15.83 μM (Figure 1f and Figure S2a) and 40.87 μM (Figure 1g and Figure S2b), respectively. CETSA is also a novel approach to determine the ability of small molecular compounds to bind to proteins. As shown in Figure 1h–j, AS caused a rightward shift in both the melting curves of GSK3α and GSK3β, further suggesting that AS could directly bind to GSK3α and GSK3β.

To investigate the effect of AS on GSK3α/β, N2a cells were treated with AS. AS was shown to reduce GSK3α and GSK3β levels in dose‐dependent (Figure 1k, *p* < 0.05) and time‐dependent manners (Figure 1l, *p* < 0.05). Consistent with the immunoblotting results, the immunofluorescence results also showed that AS reduced the fluorescence intensity of GSK3α/β in N2a cells (Figure 1m and Figure S3, *p* < 0.001). β‐catenin is an indicator of GSK3α/β enzymic activity since GSK3α/β could promote β‐catenin degradation via phosphorylating β‐catenin (Misztal et al., 2017). As expected, AS treatment significantly reduced β‐catenin (Figure 1k,l). These studies suggested that AS‐induced downregulation of GSK3α/β was accompanied by reduced GSK3α/β enzymic activity in N2a cells. Furthermore, AS also reduced GSK3α/β levels in HEK293T, BV2, HepG2, and LN229 cells (Figure S4), suggesting that the pharmacological action of AS on GSK3α/β is not specific to neurons. Additionally, in distinction from the conventional GSK3α/β inhibitors such as AR‐A014418 and SB216763, AS treatment directly reduced GSK3α/β expression rather than directly targeting enzyme activity of GSK3α/β (Figure S5). Notably, the AS‐induced reductions of GSK3α and GSK3β were completely reversed after replacement with an AS‐free medium (Figure 1n), indicating that the reduced GSK3α/β expression was directly ascribed to the AS treatment.

Given that AS is known to be an inhibitor of the transcription factor FOXO1 (Nagashima et al., 2010), we assessed whether the decreases in GSK3α and GSK3β expressions were due to their transcriptional inhibition. CHX can block the protein translation process in cells, thus permitting natural protein degradation (Fan et al., 2019). The N2a cells were treated with CHX and CHX plus AS at different time points. Our data revealed that CHX plus AS treatment significantly increased the degrading ratios of GSK3α and GSK3β and then reduced the halflife of GSK3α and GSK3β (Figure 1o–s). In addition, AS treatment did not change the mRNA levels of GSK3α and GSK3β compared to vehicle‐treated N2a cells (Figure 1t,u, *p* > 0.05), further suggesting that AS reduced GSK3α and GSK3β expressions were independent of transcriptional regulation. Of note, FOXO1 had almost no expression in N2a cells, but a high expression in MN9D cells and SH‐SY5Y cells (Figure S6a). Meanwhile, the knockdown of FOXO1 in MN9D cells did not result in downregulations of GSK3α and GSK3β (Figure S6b). These studies suggested that AS‐induced downregulations of GSK3α and GSK3β are independent of FOXO1.

### 3.2 AS treatment reduces intracellular GSK3α/β content via facilitating GSK3α/β exocytosis

**FIGURE 2:**
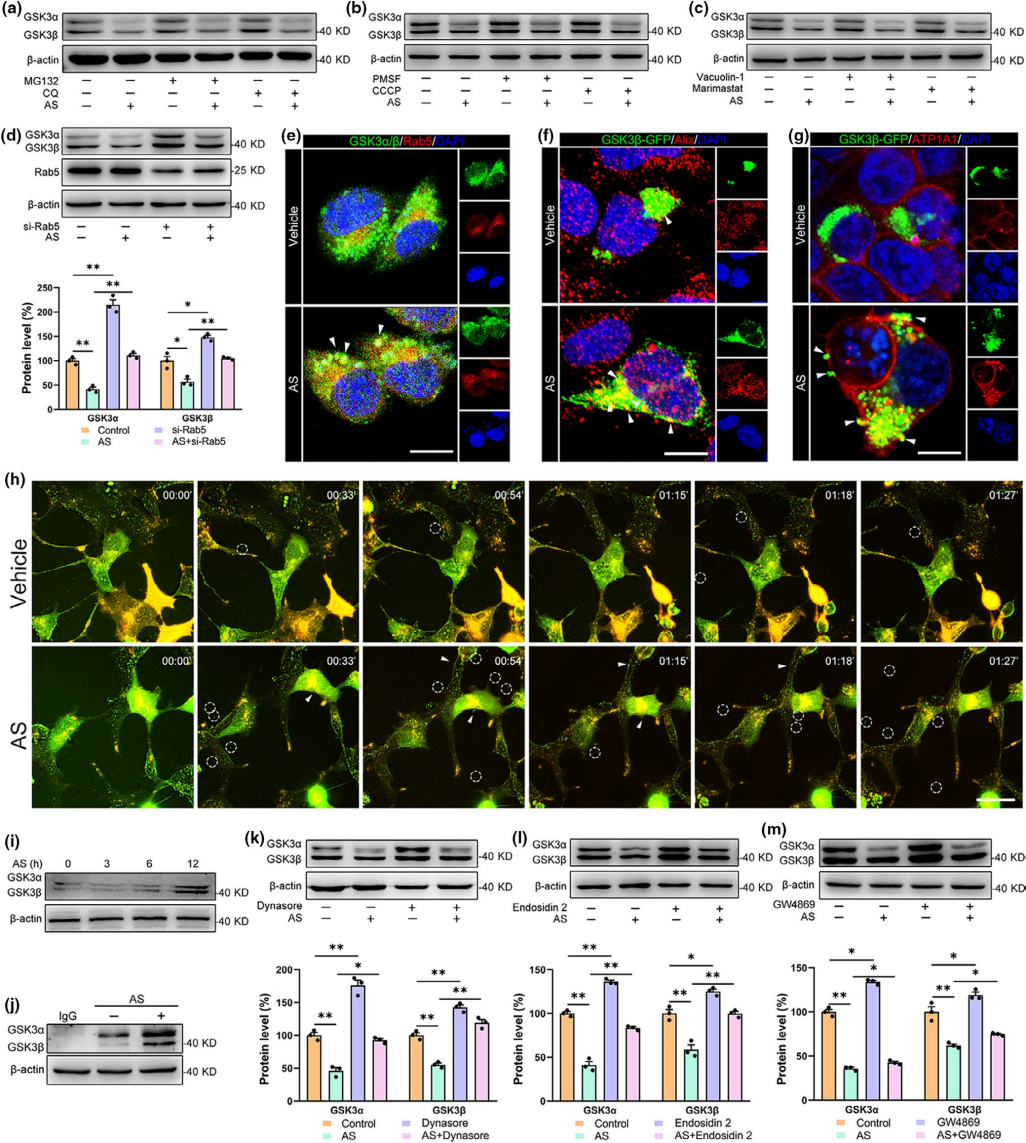
AS treatment reduces intracellular GSK3α/β content via facilitating GSK3α/β exocytosis. (a–c) The MG132 (30 nM), CQ (10 μM), CCCP (10 μM), PMSF (100 μM), Vacuolin‐1 (10 μM), and Marimastat (10 μM) treatments did not reverse the AS‐induced reduction in GSK3α/β in N2a cells. (d) Rab5 knockdown largely abrogated the AS‐mediated intracellular GSK3α/β reduction. (e) AS (0.5 μM) treatment for 2 h enhanced the co‐localization between GSK3α/β (green) and Rab5 (red) in N2a cells. The nuclei were marked by DAPI (blue). Scale bar, 10 μm. (f) Immunofluorescence images showed the increased co‐localization between GSK3α/β‐GFP (green) and MVBs marker Alix (red) in HEK293T cells overexpressing GSK3β‐GFP after AS (0.5 μM) treatment for 3 h. The nuclei were marked by DAPI (blue). Scale bar, 10 μm. (g) AS (0.5 μM) treatment for 6 h changed the distribution of GSK3β‐GFP (green) in HEK293T cells overexpressing GSK3β‐GFP. The cell membranes and nuclei were marked with ATP1A1 (red) and DAPI (blue). The white arrows showed the GSK3α/β that is being secreted. Scale bar, 15 μm. (h) Live cell imaging of HEK293T cells after overexpressing GSK3β‐GFP then AS treatment for 1 h. Cell membranes were marked with Dil (red). The white arrows indicate the GSK3β‐GFP that is being secreted. White circles showed the secreted GSK3β‐GFP. Scale bar, 15 μm. (i) The medium proteins were enrichment using acetone. Immunoblotting showed the increased content of GSK3α/β in the medium after AS (0.5 μM) treatment for 3, 6, and 12 h. The expressions of β‐actin in cell lysates as the inner control. (j) The medium proteins were enrichment using GSK3α/β‐coated beads. Immunoblotting showed an increased content of GSK3α/β in the medium after AS (0.5 μM) treatment for 12 h. The expressions of β‐actin in cell lysates as the inner control. (k, l) Dynasore (10 μM) and Endosidin 2 (0.2 mM) treatments largely abrogated the actions of AS on GSK3α/β in N2a cells. (m) GW4869 (5 μM) treatment partly reversed AS‐induced intracellular reduction in GSK3α/β in N2a cells. *n* = 3; *p < 0.05, **p < 0.01.

Protein degrading systems play key roles in protein homeostasis. To uncover the mechanism regarding AS‐induced downregulations of GSK3α and GSK3β, the inhibitors of different protein degrading systems were employed. As shown in Figure 2a–c, inhibitors for lysosome (chloroquine, CQ, *p* > 0.05), proteasome (MG132, *p* > 0.05), serine protease (PMSF, *p* > 0.05), mitochondrial uncoupling (carbonyl cyanide m‐chlorophenylhydrazone, CCCP, *p* > 0.05), matrix metalloproteinases (MMPs) (Marimastat, *p* > 0.05), and lysosomal exocytosis (Vacuolin‐1, *p* > 0.05) did not rescue the AS‐induced downregulations of GSK3α and GSK3β in N2a cells. Considering that lysosome and proteasome are the major intracellular protein degradation pathways, we extended the treatment time of CQ and MG132, and we also found no significant changes in the expression levels of GSK3α/β (Figure S7).

Rab5 is a specific marker of early endosome, which is also involved in exocytosis (Bittel & Jaiswal, 2023). Interestingly, the knockdown of Rab5 significantly increased the GSK3α and GSK3β expressions compared to the vehicle‐treated N2a cells and largely reversed the AS‐induced downregulations of GSK3α and GSK3β (Figure 2d, *p* < 0.01). Meanwhile, AS intensified the co‐localization of GSK3α/β and Rab5 in N2a cells (Figure 2e and Figure S8a, *p* < 0.05), suggesting that AS‐induced downregulations of GSK3α and GSK3β may be highly associated with the endosome‐mediated cargo transport. Lysosomal degradation or exocytosis is the predominant goal for cargoes in endosomes. Considering the lysosomal inhibitor (CQ) could not reverse the AS‐induced downregulations of GSK3α and GSK3β (Figure 2a), we suggested that AS may reduce GSK3α/β expression via promoting endosome‐mediated exocytosis. Early endosomes could further mature and transform into late endosomes, also known as multivesicular bodies (MVBs), which is a vital step for exocytosis. Indeed, the co‐localization of GSK3β and the MVBs biomarker Alix was increased after AS treatment in HEK293T cells overexpressing GSK3β‐GFP (Figure 2f and Figure S8b, *p* < 0.05) and AS treatment increased the content of GSK3α/β in the Alix‐positive vesicles from N2a cells (Figure S9). Moreover, AS treatment also caused partial green spots to seep out of the cell membrane in HEK293T cells overexpressing GSK3β‐GFP (Figure 2g and Figure S8c), indicating that AS may own the capacity to promote GSK3α/β secretion. Indeed, acetone concentrated (Figure 2i, *p* < 0.01) or immunomagnetic bead enrichment (Figure 2j, *p* < 0.01) the proteins in culture medium showed that AS treatment significantly increased the secretions of GSK3α and GSK3β from N2a cells. Moreover, live cell imaging also visualized that AS treatment increased GSK3β‐GFP secretion in HEK293T cells overexpressing GSK3β‐GFP (Figure 2h). These studies indicated that AS reduces intracellular GSK3α and GSK3β contents by promoting the secretions of GSK3α and GSK3β.

Dynasore inhibits dynamin protein and reduces the secretion of cellular vesicles (Moro et al., 2021). As shown in Figure 2k, Dynasore effectively mitigated the effects of AS in N2a cells (*p* < 0.05), suggesting that AS‐mediated secretions of GSK3α and GSK3β were in a dynamin‐dependent manner. Dynamin functions are highly related to the process of exocytosis (Bayonés et al., 2022). Indeed, the exocytosis inhibitor Endosidin 2 significantly upregulated the expression of GSK3α/β and largely blocked the AS‐induced downregulation of GSK3α/β in N2a cells (Figure 2l, *p* < 0.01). Notably, the inhibitor of exosomes, GW4869, also partly rescued the AS‐induced downregulation of GSK3α/β (Figure 2m, *p* < 0.05). These data suggested that AS reduces intracellular GSK3α/β content via promoting GSK3α/β exocytosis, and some of the GSK3α/β was loaded into the exosomes.

### 3.3 AS treatment reduces Tau hyperphosphorylation in vitro

**FIGURE 3:**
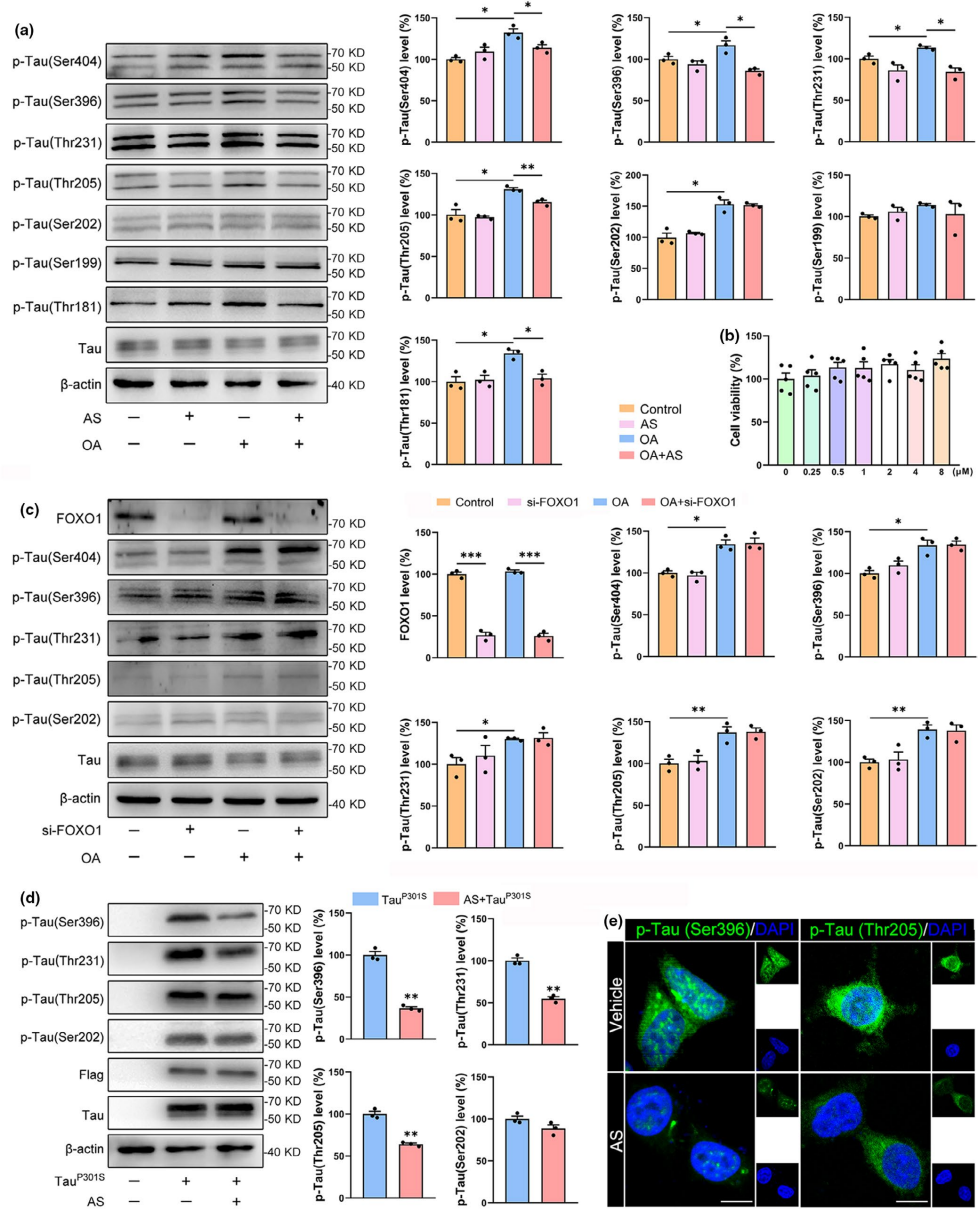
AS treatment reduces Tau hyperphosphorylation in vitro. (a) AS treatment reversed the OA‐induced Tau hyperphosphorylation at Ser404, Ser396, Thr231, Thr205, and Thr181, but not at Ser202, Ser199 in SH‐SY5Y cells. (b) Cell viability of SH‐SY5Y cells after treatment with different concentrations of AS for 24 h. (c) FOXO1 knockdown could not reverse the OA‐induced Tau hyperphosphorylation at Ser404, Ser396, Thr231, Thr205, and Ser202. (d) AS (0.5 μM) treatment for 6 h inhibited Tau hyperphosphorylation at Ser396, Thr231, and Thr205, but not at Ser202 in HEK293T cells overexpressing Tau^P301S^ mutant. (e) Immunofluorescence images showed the reduced fluorescence intensities of p‐Tau(Ser396) and p‐Tau(Thr205) in AS‐treated HEK293T cells overexpressing Tau^P301S^ mutant. Scale bar, 10 μm. *n* = 3; *p < 0.05, **p < 0.01, ***p < 0.001.

To investigate whether AS can affect the phosphorylation of Tau protein by decreasing GSK3α/β, Tau hyperphosphorylation was induced by Okadaic Acid (OA) and further treated with AS in SH‐SY5Y cells. AS did not cause any cytotoxicity to SH‐SY5Y cells (Figure 3b). Meanwhile, AS treatment reversed the OA‐induced Tau hyperphosphorylation at Ser404, Ser396, Thr231, Thr205, and Thr181, but not at Ser202 and Ser199 (Figure 3a). Considering FOXO1 was highly expressed in SH‐SY5Y cells, to rule out that FOXO1 may involve in the AS‐induced inhibition of Tau hyperphosphorylation, FOXO1 was silenced in SH‐SY5Y cells. As shown in Figure 3c, FOXO1 knockdown could not reverse the OA‐induced Tau hyperphosphorylation in SH‐SY5Y cells, suggesting that AS‐induced downregulation of Tau hyperphosphorylation was independent of FOXO1. Furthermore, the Tau^P301S^ mutate was introduced in the HEK293T cells, and AS treatment also inhibited Tau hyperphosphorylation at Ser396, Thr231, and Thr205 but not Ser202 (Figure 3d). Immunostaining also visualized the downregulations of p‐Tau (Ser396) and p‐Tau (Thr205) after AS treatment in HEK293T cells overexpressing Tau^P301S^ mutate (Figure 3e and Figure S10, *p* < 0.01). Then, we overexpressed GSK3β‐GFP in the Tau^P301S^‐HEK293T cells (Figure S11a). We found that overexpression of GSK3β‐GFP led to increased levels of p‐Tau (Ser396), p‐Tau (Thr205), and p‐Tau (Ser199), and treatment with AS significantly reversed this circumstance (Figure S11b, *p* < 0.05). These data suggested that AS inhibited Tau hyperphosphorylation by targeting GSK3α/β.

### 3.4 AS penetrates the BBB and reduces GSK3α/β expression in P301S transgenic mice

**FIGURE 4:**
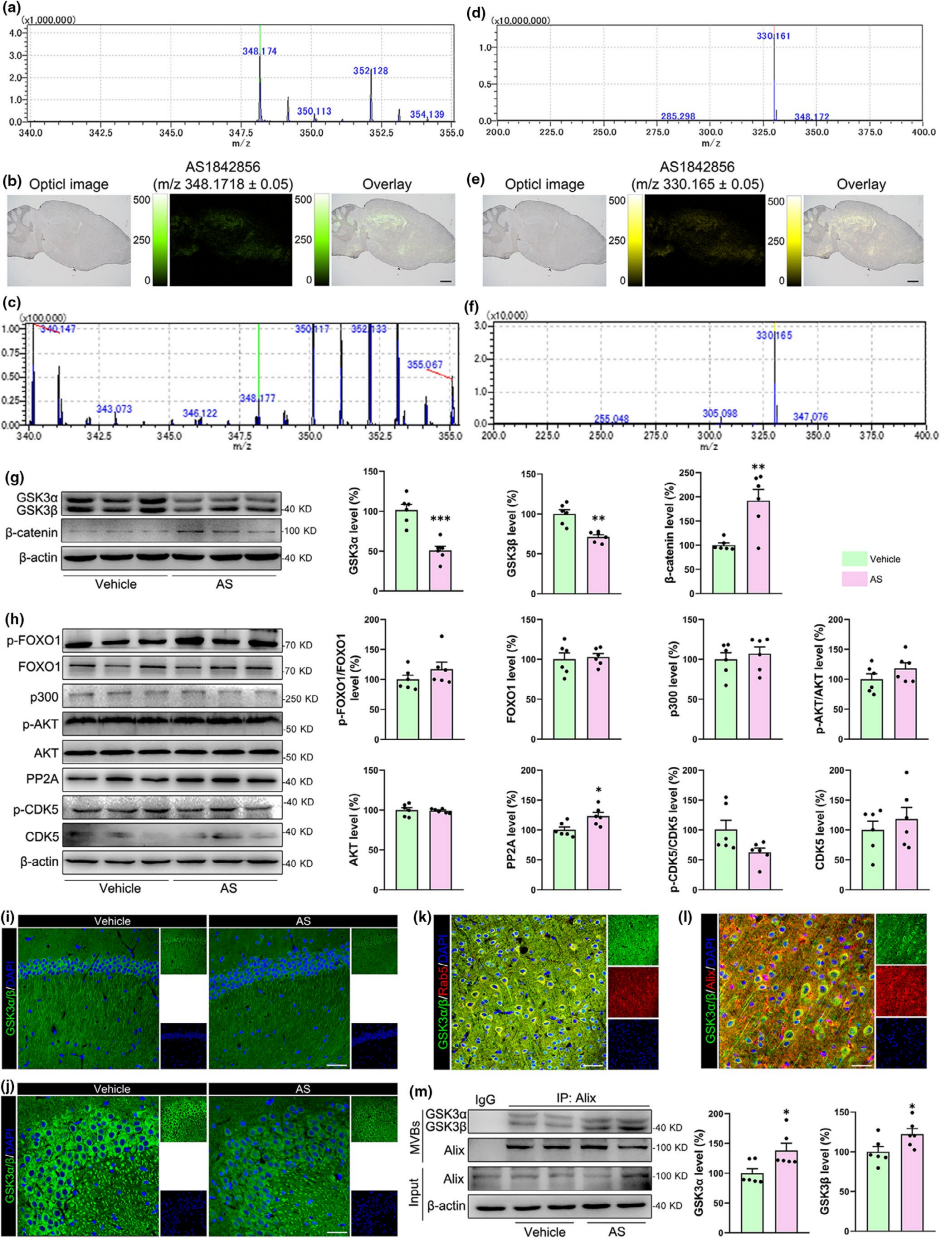
AS penetrates the BBB and reduces GSK3α/β expression in P301S transgenic mice. (a–f) MS imaging (a–c) and MS/MS imaging (d–f) for the distribution of AS within regions of the brain. The structures and mass spectrum of AS (a). The corresponding MS/MS profiles of reference standards of AS (d). MS ion images representing spatial distributions of AS (m/z 348.1718 ± 0.05) within regions of the brain (b) and MS/MS imaging for distribution of AS within regions of the brain (e). All ion images were normalized to the CHCA matrix signal (pixel size, 45 μm). MALDI‐MSI (c) and MALDI‐MS/MS (f) single pixel mass spectra from the brain. *n* = 3. (g) Immunoblotting evaluated the expressions of β‐catenin and GSK3α/β in the cortex of P301S mice after treatment with AS for 8 weeks. *n* = 6. (h) Immunoblotting evaluated the expressions of FOXO1, p‐FOXO1, PP2A, p300, AKT, CDK5, and p‐CDK5 in the cortex of P301S mice after treatment with AS for 8 weeks. *n* = 6. (i, j) Immunofluorescence images show the decreased fluorescence intensities of GSK3α/β (green) in the CA1 (i) and CA3 (j) regions of the hippocampus. *n* = 6; Scale bar, 100 μm. (k) GSK3α/β (green) was co‐localized with Rab5 (red) in P301S transgenic mouse cortex. *n* = 6; Scale bar, 100 μm. (l) GSK3α/β (green) was co‐localized with Alix (red) in P301S transgenic mouse cortex. *n* = 6; Scale bar, 100 μm. (m) AS treatment increased the content of GSK3α/β in the vesicles from the P301S mouse cortex. *n* = 6. *p < 0.05, **p < 0.01, ***p < 0.001.

The BBB permeability is vital for drugs to enter the brain parenchyma. Mass spectrometry imaging is a new technology for displaying drug distribution at the tissue in situ level (Zhao et al., 2023). Our study showed that AS could effectively penetrate the brain parenchyma, as evidenced by both the MS (Figure 4a–c) and MS/MS (Figure 4d–f) imaging of brain sections from mice after AS intraperitoneal injection using imaging mass microscope (iMScope). Similarly, the signals of AS were also detected in the liver (Figure S12a–e), spleen (Figure S12f–j), and pancreas (Figure S12k–o). These data showed that AS could penetrate the BBB and accumulate in the peripheral organs. Subsequently, we used 4‐month‐old male P301S transgenic mice (Xu et al., 2014) and treated them with AS for 8 weeks. Immunoblotting studies showed that AS treatment significantly reduced GSK3α/β levels (*p* < 0.01), and was accompanied by increased β‐catenin expression, suggesting that AS treatment inhibited GSK3α/β bioactivity via downregulating GSK3α/β protein expression (Figure 4g). Meanwhile, AS demonstrated notable efficacy in significantly reducing GSK3α/β levels across various peripheral organs, such as the liver, spleen, and kidneys, without inducing significant cytotoxic effects (Figure S13). Moreover, AS treatment increased PP2A expression (*p* < 0.05) but did not influence the expressions of p‐FOXO1, FOXO1, CDK5, p‐CDK5, AKT, p‐AKT, and p300 (Figure 4h). Immunostaining studies also showed that the expressions of GSK3α/β in both the CA1 region and CA3 region of the hippocampus were decreased after AS treatment (Figure 4i,j, and Figure S15). Furthermore, the GSK3α/β was co‐localized with Rab5 (Figure 4k) and Alix (Figure 4l) in P301S transgenic mice, and AS treatment significantly increased the content of GSK3α/β in the vesicles from P301S transgenic mouse brains (Figure 4m, *p* < 0.05). These studies suggested that AS treatment could also reduce GSK3α/β expression in vivo by promoting GSK3α/β exocytosis.

### 3.5 AS treatment inhibits Tau hyperphosphorylation and improves cognitive capacity in P301S transgenic mice

**FIGURE 5:**
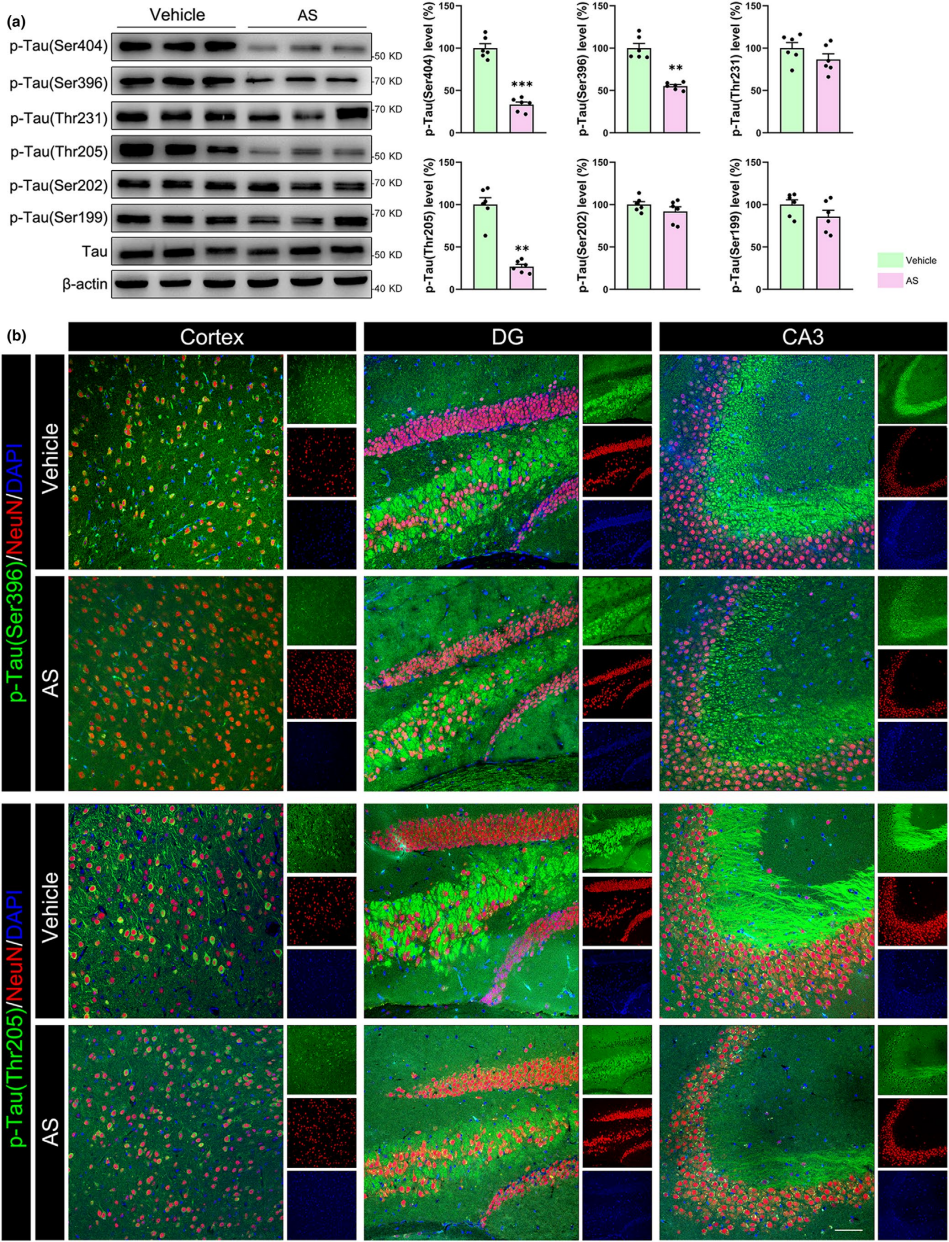
AS treatment inhibits Tau hyperphosphorylation in the P301S transgenic mouse brains. (a) AS treatment reduced the Tau hyperphosphorylation at Ser404, Ser396 and Thr205, but not at Thr231, Ser202, and Ser199 in P301S transgenic mouse cortex. *n* = 6. (b) Immunofluorescence images showed the reduced fluorescence intensities of p‐Tau(Ser396) and p‐Tau(Thr205) in the cortex, DG and CA3 regions of the hippocampus in P301S transgenic mice. *n* = 6; Scale bars, 200 μm. **p < 0.01, ***p < 0.001.

**FIGURE 6:**
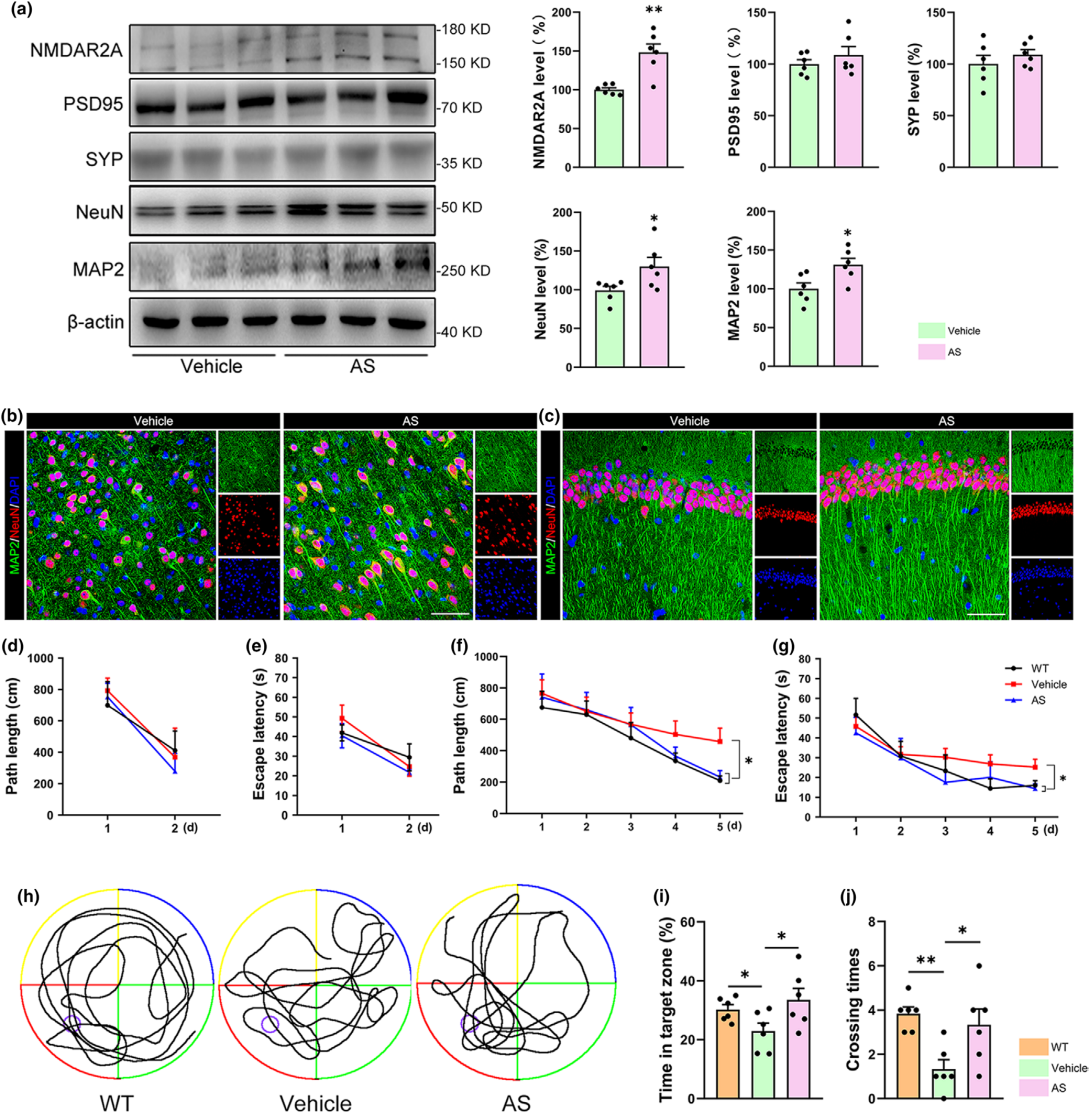
AS treatment inhibits neuronal loss and improves cognitive function in P301S transgenic mice. (a) Immunoblotting detected the expression levels of NMDAR2A, PSD95, SYP, NeuN, and MAP2 in P301S transgenic mouse hippocampus after AS treatment. *n* = 6. (b, c) AS treatment elevated the fluorescence intensities of MAP2 (green) in the cortex and CA1 region of the hippocampus. *n* = 6; Scale bars, 100 μm. (d, e) The path lengths and escape latencies to visit the visible platform in the three indicated groups of mice. (f, g) The path lengths and escape latencies to visit the hidden platform in the three indicated groups of mice. (h) The typical path maps of the mice in the probe trial tests. (i) Statistical graph of time spent in target zone in the probe trial tests. (j) Statistical graph of crossing times in the probe trial tests. *n* = 6. *p < 0.05, **p < 0.01.

Immunoblotting studies showed that AS treatment reduced the levels of p‐Tau(Ser404), p‐Tau(Ser396), and p‐Tau(Thr205), but not p‐Tau(Ser 202), p‐Tau(Thr231), and p‐Tau(Ser 199) (Figure 5a). Immunostaining further confirmed the reduced expressions of p‐Tau(Ser396) and p‐Tau(Thr205) in both the cortex and hippocampus from AS‐treated P301S transgenic mice (Figure 5b and Figure S16). Tau hyperphosphorylation impairs synaptic plasticity and promotes neuroinflammation and neuronal loss during the process of AD. Our studies showed that AS treatment increased the protein levels of N‐methyl‐D‐aspartate (NMDA) receptors 2A (NMDAR2A) (*p* < 0.01), microtubule‐associated protein 2 (MAP2) (*p* < 0.05), and neuronal nuclei (NeuN) (*p* < 0.05), but not the postsynaptic density protein 95 (PSD95) and synaptophysin (SYP) (Figure 6a). The increased expression of MAP2 in cortex and CA1 region of P301S transgenic mice were also verified by immunostaining (Figure 6b,c and Figure S17), suggesting that AS treatment may facilitate neuronal survival in P301S transgenic mice. In addition, AS treatment considerably decreased the expression of tumor necrosis factor‐α (TNF‐α) (Figure S18, *p* < 0.01), indicating that AS may also inhibit neuroinflammation in P301S transgenic mice.

The neuropathological changes are highly associated with cognitive alternation. The spatial learning and memory capacities of the mice were assessed using the Morris water maze test. The path length (Figure 6d, *p* > 0.05) and escape latencies (Figure 6e, *p* > 0.05) in the visible platform tests were similar between the wild‐type (WT) mice, vehicle‐treated P301S transgenic mice, and AS‐treated P301S transgenic mice, indicating that there is no difference in the motor ability and visual acuity of the indicated three groups of mice. In the hidden platform tests, the vehicle‐treated P301S transgenic mice had a longer path length (Figure 6f, *p* < 0.05) and escape latency (Figure 6g, *p* < 0.05) compared to the AS‐treated P301S transgenic mice and WT mice. In the probe trial test, the AS‐treated P301S transgenic mice showed more crossing times and time spent in the target zone than vehicle‐treated P301S transgenic mice (Figure 6h–j, *p* < 0.05). These data indicated that AS improves the learning and memory abilities of P301S transgenic mice. We also tested the anxiety‐like behavior of the mice using open field test. However, AS treatment could not alleviate the anxiety‐like behavior in P301S transgenic mice (Figure S19).

## 4 DISCUSSION

GSK3α/β is intimately associated with the pathologic progression of AD (Behl et al., 2023); thus, an increasing number of studies are focused on the expansion of the GSK3α/β inhibitors. However, the side effects and low BBB permeability hinder the applications of GSK3α/β inhibitors (Li et al., 2023). In this study, we first demonstrated that AS can directly bind to GSK3α/β to accelerate GSK3α/β exocytosis, and effectively reduce intracellular GSK3α/β levels in vitro and in vivo. Furthermore, AS is a small molecular compound that has the characteristic of crossing the BBB. In addition, AS can effectively inhibit the Tau hyperphosphorylation and improve cognitive capacity in P301S transgenic mice. Hence, AS may be a potential drug for treating AD and Tau hyperphosphorylation‐related diseases.

The exploration of GSK3α/β inhibitors for the treatment of AD is a potential therapeutic avenue. Lateral ventricular injection of GSK3α/β inhibitor SB216763 has confirmed the beneficial effects of reduced GSK3β activity in AD models (Lauretti et al., 2020). Conversely, GSK3α/β activation exacerbates AD pathology symptoms (Terwel et al., 2008; Wang et al., 2017). Although some advancements were achieved during the experimental phase for GSK3α/β inhibitors, most of the inhibitors failed in animal tests or clinical trials. For example, LY2090314 and AZD1080 were discontinued due to limited clinical benefit and safety concerns such as chronic cholecystitis (Bhat et al., 2018; Rizzieri et al., 2016). Furthermore, Tideglusib, as a non‐ATP‐competitive GSK3β inhibitor, reduced Tau phosphorylation levels, decreased Aβ deposition and astrocyte proliferation, and improved memory performance in the AD mouse model (Sereno et al., 2009; Wang et al., 2016). This drug has reached phase II clinical trials with no reported safety events, but the drug did not achieve the primary endpoint in patients with AD and PSP (Bhat et al., 2018; Tolosa et al., 2014). In this study, we identified that AS could potently inhibit the protein expression of GSK3α/β, which is different from the existing GSK3α/β inhibitors that just repress the activity of GSK3α/β. Compared to the other GSK3α/β inhibitors, the different mechanisms regarding AS on GSK3α/β may lead to different drug tolerance and therapeutic effects in vivo. Moreover, AS could penetrate BBB and accumulate in the hippocampus and cortex, thereby reducing GSK3α/β expression and Tau hyperphosphorylation in P301S transgenic mice. Hence, AS may be a potential drug for treating Tau hyperphosphorylation‐related diseases.

Currently, most studies on GSK3 were focused on GSK3β, but disregarded the function of GSK3α. Previous studies revealed that GSK3β knockout mice suffered embryonic lethality, while GSK3α didn't (Kaidanovich‐Beilin et al., 2009; Kerkela et al., 2008), indicating the different physiological functions of GSK3α and GSK3β. Indeed, GSK3α but not GSK3β is required for NMDAR‐dependent long‐term depression (LTD) in the hippocampus (Draffin et al., 2021). GSK3β is an important enzyme for Tau hyperphosphorylation, while GSK3α also promotes Tau hyperphosphorylation in AD pathology and normal physiologic conditions (Dunning et al., 2016; Maurin et al., 2013). In this study, we found that AS was able to directly bind and subsequently reduce both GSK3α and GSK3β in vitro and in vivo. Interestingly, we found that AS bound GSK3α better than GSK3β in the BLI assay, and correspondingly, AS reduced GSK3α more significantly. Moreover, the binding and dissociation curves of AS with GSK3α and GSK3β in the BLI assay (Figure S2) exerted a more rapid binding and dissociation of GSK3α with AS. However, in the CETSA, we found that AS caused a more pronounced rightward shift of the GSK3β melting curve. These results suggested that the binding of AS to GSK3α was more rapid but not as stabilized as GSK3β.

The mechanism concerning GSK3α/β protein reduction is rarely reported. Based on the GSK3α/β inhibitor AZD2858, the proteolysis targeting chimeras (PROTACs) PT‐65 was synthetized. It significantly inhibited GSK3α/β expression via promoting GSK3α/β degradation by ubiquitination pathway (Qu et al., 2021). However, our study showed that GSK3α/β could be not degraded by both the lysosome and proteasome under normal physiological conditions as the lysosomal inhibitor (CQ) and proteasomal inhibitor (MG132) did not increase the GSK3α/β expression. Similarly, inhibitions of serine protease, mitochondrial uncoupling, MMPs, and lysosomal exocytosis also could not increase the expression of GSK3α/β, suggesting that these protein clearance systems did not involved in the modulation of GSK3α/β homeostasis under normal physiological conditions. Previous studies revealed that Rab cascade signaling mediates the transport of secretory vesicles from the trans‐Golgi network to the plasma membrane (Borchers et al., 2021). Interestingly, Rab5 was co‐localized with GSK3α/β, and interfering with intracellular cargo transport by knocking down Rab5 increased GSK3α/β expression and reversed the AS‐mediated GSK3α/β reduction, indicating that the GSK3α/β may be naturally secreted by the Rab5‐positive vehicle. Given that drugs can induce alterations in protein conformation (Guo et al., 2022; Wang et al., 2023), we suggested that AS may induce GSK3α/β secretion through promoting GSK3α/β translocation to the Rab5‐positive vesicles by conformational shifts in GSK3α/β protein. This intriguing possibility will be a focal point of our future investigations. Dynamin and F‐actin mediate intracellular vesicle release during the process of exocytosis (Bayonés et al., 2022), and secretion of cytosolic vesicles can be inhibited by Dynasore (dynamin inhibitor) (Moro et al., 2021). Indeed, Dynasore treatment increased GSK3α/β expression and reversed the AS‐induced reduction in GSK3α/β. Similar results also were observed after treatment with an exocytosis inhibitor and exosome inhibitor. In addition, AS treatment considerably elevated the GSK3α/β content in the cell culture medium. These results suggested that the intracellular GSK3α/β is predominantly secreted to the extracellular environment, and AS could directly bind to GSK3α/β to accelerate GSK3α/β secretion. Consistent with our observations, previous studies also showed that GSK3α/β could be sequestered into the multivesicular endosomes to reduce its activity (Taelman et al., 2010). Furthermore, the exosome secretion of GSK3β was also observed after AS treatment in naïve CD4^+^ T cells, and the authors suggested that this effect was ascribed to the FOXO1 inhibition but not the direct action of AS (J. Jin et al., 2020). However, in this study, we found that FOXO1 was almost not expressed in N2a cells, whereas AS treatment still promoted GSK3α/β exocytosis; furthermore, FOXO1 knockdown could not reduce the intracellular GSK3α/β level in the MN9D cells that highly expressing FOXO1. These results remind us that AS‐mediated intracellular GSK3α/β reduction is independent of FOXO1. Hence, AS may be a dual inhibitor of both FOXO1 and GSK3α/β. Currently, numerous investigations utilize AS as a specific FOXO1 inhibitor (Eng et al., 2023; Nagashima et al., 2010). Based on this study, we propose that when employing AS for future studies, the AS‐mediated GSK3α/β reduction should be considered.

Given that AS could directly inhibit the intracellular GSK3α/β content, we conjectured that AS may own the ability to alleviate Tau pathology. Similar to our previous study (Zhao et al., 2023), the iMScope effectively visualized the AS biodistribution at the tissue in situ level. Although most of the AS was accumulated in the liver and spleen, a certain amount of AS was observed in the brain tissue, indicating that AS could penetrate the BBB. As expected, AS inhibited the intracellular GSK3α/β level, accompanied by the sharp reduction in Tau phosphorylation. Increased PP2A was shown to inhibit Tau hyperphosphorylation (Saletti et al., 2023). AS treatment also upregulated PP2A expression in P301S transgenic mice. In line with our observation, previous studies uncovered that activation or overexpression of GSK3β caused a decrease in PP2A levels (Jin et al., 2019; Yao et al., 2012), while inhibition of GSK3β increased PP2A levels (Yao et al., 2012). Furthermore, despite AS's ability to augment PP2A content in N2a cells, CETSA analysis revealed no discernible binding affinity between AS and PP2A (Figure S14). These results indicated that AS may also promote PP2A expression via inhibiting intracellular GSK3β content, which subsequently inhibited Tau hyperphosphorylation. Notably, AS is a specific inhibitor of FOXO1 (Nagashima et al., 2010). However, long‐term treatment of AS did not influence the phosphorylation of FOXO1 at Ser256 which was widely used to evaluate the activity of FOXO1 (Pandey et al., 2023). This circumstance may be ascribed to the low content of AS in the brain tissue. It is worth noting that FOXO1 activation or overexpression reduced Tau phosphorylation in vitro (Zhang et al., 2020, 2024). However, FOXO1 knockdown could not change the Tau phosphorylation in our study. This circumstance may be attributed to the different cell models of Tau hyperphosphorylation. Nevertheless, AS inhibited the Tau hyperphosphorylation in vitro and in vivo. Hence, we may conclude that AS‐inhibited Tau hyperphosphorylation is ascribed to the AS‐induced intracellular GSK3α/β downregulation but not the inhibition of FOXO1 activity.

In summary, we first clarify that the intracellular GSK3α/β homeostasis is dependent on its exocytosis. Furthermore, AS could directly bind to GSK3α/β to reduce intracellular GSK3α/β content by accelerating GSK3α/β exocytosis. In addition, AS inhibited Tau hyperphosphorylation by suppressing GSK3α/β expression in P301S transgenic mice. Hence, AS is a GSK3α/β‐targeting compound that could be considered for Tau hyperphosphorylation‐related disease.

## AUTHOR CONTRIBUTIONS

Da‐Long He performed most of the experiments and wrote the manuscript; Xiao‐Yu Zhang, Jing‐Yang Su, Qi Zhang, Ling‐Xiao Zhao, Ting‐Yao Wu, Hang Ren, Rong‐Jun Jia, Xian‐Fang Lei, Wen‐Jia Hou, and Wen‐Ge Sun contributed to experiments; Yong‐Gang Fan and Zhan‐You Wang designed the experiments and edited the manuscript. All authors have read and approved the final manuscript.

## FUNDING INFORMATION

This study was financially supported by the National Natural Science Foundation of China (82301626, 82371447), the Science and Technology Program of Liaoning Province (2023‐BS‐095), and the Liaoning Science and Technology Innovation Leading Talent Project (XLYC2002016).

## CONFLICT OF INTEREST STATEMENT

None declared.

## Supporting information


Table S1

Tables S2–S3

Appendix S1

## Data Availability

Data will be made available on request.
