# Supplementary material for: Identification of AS1842856 as a novel small‐molecule GSK3α/β inhibitor against Tauopathy by accelerating GSK3α/β exocytosis
Source: Aging Cell. 2024 Sep 17;24(1):e14336. doi: 10.1111/acel.14336 (PMC11709109; doi:10.1111/acel.14336)
Supplement: Supplementary file 1 — Table S1 [file ACEL-24-e14336-s001.pdf]

SwissTargetPrediction

Target	Common name	Uniprot ID	ChEMBL ID	Target Class	Probability*	Known actives (3D/2D)
Glycogen synthase kinase-3 beta	GSK3B	P49841	CHEMBL262	Kinase	0.691501945475	83 / 55
Protein farnesyltransferase	FNTA<br>FNTB	P49354<br>P49356	CHEMBL2094108	Enzyme	0.0972399887602	63 / 0
Anandamide amidohydrolase	FAAH	O00519	CHEMBL2243	Enzyme	0.0972399887602	20 / 0
Phospholipase A2 group IIA	PLA2G2A	P14555	CHEMBL3474	Enzyme	0.0972399887602	88 / 0
G protein-coupled receptor 44	PTGDR2	Q9Y5Y4	CHEMBL5071	Family A G protein-coupled receptor	0.0972399887602	1191 / 0
Prostanoid EP2 receptor	PTGER2	P43116	CHEMBL1881	Family A G protein-coupled receptor	0.0972399887602	123 / 0
Nuclear receptor subfamily 4 group A member 1	NR4A1	P22736	CHEMBL1293229	Nuclear receptor	0.0972399887602	5 / 0
Carnitine O-palmitoyltransferase 1, liver isoform	CPT1A	P50416	CHEMBL1293194	Enzyme	0.0972399887602	168 / 0
L-lactate dehydrogenase A chain	LDHA	P00338	CHEMBL4835	Enzyme	0.0972399887602	11 / 0
Integrin alpha-4/beta-1	ITGB1<br>ITGA4	P05556<br>P13612	CHEMBL1907599	Membrane receptor	0.0972399887602	414 / 0
Peroxisome proliferator-activated receptor alpha	PPARA	Q07869	CHEMBL239	Nuclear receptor	0.0972399887602	263 / 0
Lysine-specific demethylase 5A	KDM5A	P29375	CHEMBL2424504	Eraser	0.0972399887602	8 / 0
Lysine-specific demethylase 5B	KDM5B	Q9UGL1	CHEMBL3774295	Eraser	0.0972399887602	7 / 0
Lysine-specific demethylase 2B	KDM2B	Q8NHM5	CHEMBL3779760	Eraser	0.0972399887602	3 / 0
Lysine-specific demethylase 4C	KDM4C	Q9H3R0	CHEMBL6175	Eraser	0.0972399887602	23 / 0
Caspase-1	CASP1	P29466	CHEMBL4801	Protease	0.0972399887602	107 / 0
L-lactate dehydrogenase B chain	LDHB	P07195	CHEMBL4940	Enzyme	0.0972399887602	6 / 0
Prostanoid EP1 receptor	PTGER1	P34995	CHEMBL1811	Family A G protein-coupled receptor	0.0972399887602	329 / 0
Aldose reductase	AKR1B1	P15121	CHEMBL1900	Enzyme	0.0972399887602	318 / 0
Cytochrome P450 26B1	CYP26B1	Q9NR63	CHEMBL3713687	Cytochrome P450	0.0972399887602	11 / 0
Cytochrome P450 26A1	CYP26A1	O43174	CHEMBL5141	Cytochrome P450	0.0972399887602	28 / 0
Retinoic acid receptor beta	RARB	P10826	CHEMBL2008	Nuclear receptor	0.0972399887602	71 / 0
Retinoic acid receptor alpha	RARA	P10276	CHEMBL2055	Nuclear receptor	0.0972399887602	72 / 0
MAP kinase signal-	MKNK2	Q9HBH9	CHEMBL4204	Kinase	0.0972399887602	19 / 0

Target	Common name	Uniprot ID	ChEMBL ID	Target Class	Probability*	Known actives (3D/2D)
integrating kinase 2						
Phosphodiesterase 4D	PDE4D	Q08499	CHEMBL288	Phosphodiesterase	0.0972399887602	90 / 0
Chymase	CMA1	P23946	CHEMBL4068	Protease	0.0972399887602	105 / 0
Carboxypeptidase B	CPB1	P15086	CHEMBL2552	Protease	0.0972399887602	14 / 0
Cathepsin G	CTSG	P08311	CHEMBL4071	Protease	0.0972399887602	15 / 0
Proto-oncogene tyrosine-protein kinase MER	MERTK	Q12866	CHEMBL5331	Kinase	0.0972399887602	2 / 0
Sodium channel protein type X alpha subunit (by homology)	SCN10A	Q9Y5Y9	CHEMBL5451	Voltage-gated ion channel	0.0972399887602	21 / 0
Carnitine O-palmitoyltransferase 1, muscle isoform	CPT1B	Q92523	CHEMBL2216739	Group translocator	0.0972399887602	113 / 0
Matrix metalloproteinase 13	MMP13	P45452	CHEMBL280	Protease	0.0972399887602	104 / 0
Matrix metalloproteinase 3	MMP3	P08254	CHEMBL283	Protease	0.0972399887602	93 / 0
Carnitine palmitoyltransferase 2	CPT2	P23786	CHEMBL3238	Enzyme	0.0972399887602	57 / 0
Matrix metalloproteinase 2	MMP2	P08253	CHEMBL333	Protease	0.0972399887602	178 / 0
Hydroxycarboxylic acid receptor 2	HCAR2	Q8TDS4	CHEMBL3785	Family A G protein-coupled receptor	0.0972399887602	29 / 0
Metabotropic glutamate receptor 2 (by homology)	GRM2	Q14416	CHEMBL5137	Family C G protein-coupled receptor	0.0972399887602	9 / 0
Integrin alpha-V/ beta-3	ITGAV ITGB3	P06756 P05106	CHEMBL1907598	Membrane receptor	0.0972399887602	190 / 0
Serine/threonine-protein kinase NEK2	NEK2	P51955	CHEMBL3835	Kinase	0.0972399887602	15 / 0
Liver glycogen phosphorylase	PYGL	P06737	CHEMBL2568	Enzyme	0.0972399887602	105 / 0
Glycogen synthase kinase-3 alpha	GSK3A	P49840	CHEMBL2850	Kinase	0.0972399887602	20 / 0
Matrix metalloproteinase 9	MMP9	P14780	CHEMBL321	Protease	0.0972399887602	88 / 0
Lysophosphatidic acid receptor Edg-4	LPAR2	Q9HBW0	CHEMBL3724	Family A G protein-coupled receptor	0.0972399887602	10 / 0
Matrix metalloproteinase 14	MMP14	P50281	CHEMBL3869	Protease	0.0972399887602	39 / 0
Matrix metalloproteinase 12	MMP12	P39900	CHEMBL4393	Protease	0.0972399887602	49 / 0
Matrix metalloproteinase 8	MMP8	P22894	CHEMBL4588	Protease	0.0972399887602	92 / 0
Aldo-keto-reductase family 1 member C3	AKR1C3	P42330	CHEMBL4681	Enzyme	0.0972399887602	132 / 0

Target	Common name	Uniprot ID	ChEMBL ID	Target Class	Probability*	Known actives (3D/2D)
Hepatocyte nuclear factor 4-alpha	HNF4A	P41235	CHEMBL5398	Unclassified protein	0.0972399887602	16 / 0
Transient receptor potential cation channel subfamily M member 8	TRPM8	Q7Z2W7	CHEMBL1075319	Voltage-gated ion channel	0.0972399887602	78 / 0
Integrin alpha-4/ beta-7	ITGB7 ITGA4	P26010 P13612	CHEMBL2095184	Membrane receptor	0.0972399887602	107 / 0
Epoxide hydratase	EPHX2	P34913	CHEMBL2409	Protease	0.0972399887602	72 / 0
Carboxypeptidase B2 isoform A	CPB2	Q96IY4	CHEMBL3419	Protease	0.0972399887602	9 / 0
Monocarboxylate transporter 1 (by homology)	SLC16A1	P53985	CHEMBL4360	Electrochemical transporter	0.0972399887602	24 / 0
ADAM17	ADAM17	P78536	CHEMBL3706	Protease	0.0972399887602	42 / 0
5-lipoxygenase activating protein	ALOX5AP	P20292	CHEMBL4550	Other cytosolic protein	0.0972399887602	69 / 0
Angiotensin-converting enzyme	ACE	P12821	CHEMBL1808	Protease	0.0972399887602	311 / 0
11-beta-hydroxysteroid dehydrogenase 1	HSD11B1	P28845	CHEMBL4235	Enzyme	0.0972399887602	52 / 0
Epidermal growth factor receptor erbB1	EGFR	P00533	CHEMBL203	Kinase	0.0972399887602	38 / 0
Thromboxane A2 receptor	TBXA2R	P21731	CHEMBL2069	Family A G protein-coupled receptor	0.0972399887602	293 / 0
Group IIE secretory phospholipase A2	PLA2G2E	Q9NZK7	CHEMBL2154	Enzyme	0.0972399887602	9 / 0
Group IIF secretory phospholipase A2	PLA2G2F	Q9BZM2	CHEMBL4278	Enzyme	0.0972399887602	6 / 0
Group IID secretory phospholipase A2	PLA2G2D	Q9UNK4	CHEMBL4281	Enzyme	0.0972399887602	5 / 0
Group X secretory phospholipase A2	PLA2G10	O15496	CHEMBL4342	Enzyme	0.0972399887602	12 / 0
Phospholipase A2 group 1B (by homology)	PLA2G1B	P04054	CHEMBL4426	Enzyme	0.0972399887602	51 / 0
Arachidonate 5-lipoxygenase	ALOX5	P09917	CHEMBL215	Oxidoreductase	0.0972399887602	94 / 0
Steryl-sulfatase	STS	P08842	CHEMBL3559	Enzyme	0.0972399887602	14 / 0
11-beta-hydroxysteroid dehydrogenase 2	HSD11B2	P80365	CHEMBL3746	Enzyme	0.0972399887602	8 / 0
Cyclooxygenase-2	PTGS2	P35354	CHEMBL230	Oxidoreductase	0.0972399887602	130 / 0
Protein-tyrosine phosphatase 1B	PTPN1	P18031	CHEMBL335	Phosphatase	0.0972399887602	95 / 0
Phospholipase A2 group V	PLA2G5	P39877	CHEMBL4323	Enzyme	0.0972399887602	8 / 0
Replication protein A 70 kDa DNA-binding subunit	RPA1	P27694	CHEMBL1764940	Unclassified protein	0.0972399887602	14 / 0

Target	Common name	Uniprot ID	ChEMBL ID	Target Class	Probability*	Known actives (3D/2D)
Neprilysin (by homology)	MME	P08473	CHEMBL1944	Protease	0.0972399887602	236 / 0
Insulin-like growth factor I receptor	IGF1R	P08069	CHEMBL1957	Kinase	0.0972399887602	11 / 0
Free fatty acid receptor 1	FFAR1	O14842	CHEMBL4422	Family A G protein-coupled receptor	0.0972399887602	159 / 0
Transforming protein RhoA	RHOA	P61586	CHEMBL6052	Unclassified protein	0.0972399887602	5 / 0
Peptidyl-prolyl cis-trans isomerase NIMA-interacting 1	PIN1	Q13526	CHEMBL2288	Enzyme	0.0972399887602	53 / 0
Cathepsin (V and K)	CTSV	O60911	CHEMBL3272	Protease	0.0972399887602	8 / 0
Intercellular adhesion molecule (ICAM-1), Integrin alpha-L/beta-2	ITGAL ICAM1 ITGB2	P20701 P05362 P05107	CHEMBL2096661	Membrane receptor	0.0972399887602	42 / 0
PI3-kinase p110-beta subunit	PIK3CB	P42338	CHEMBL3145	Enzyme	0.0972399887602	18 / 0
PI3-kinase p110-alpha subunit	PIK3CA	P42336	CHEMBL4005	Enzyme	0.0972399887602	25 / 0
Gamma-amino-N-butyrate transaminase (by homology)	ABAT	P80404	CHEMBL2044	Transferase	0.0972399887602	4 / 0
Steroid 5-alpha-reductase 2	SRD5A2	P31213	CHEMBL1856	Oxidoreductase	0.0972399887602	50 / 0
Bile acid receptor FXR	NR1H4	Q96RI1	CHEMBL2047	Nuclear receptor	0.0972399887602	62 / 0
Peroxisome proliferator-activated receptor delta	PPARD	Q03181	CHEMBL3979	Nuclear receptor	0.0972399887602	144 / 0
CREB-binding protein/p53	CREBBP	Q92793	CHEMBL5747	Writer	0.0972399887602	10 / 0
Matrix metalloproteinase 1	MMP1	P03956	CHEMBL332	Protease	0.0972399887602	49 / 0
Retinoic acid receptor gamma	RARG	P13631	CHEMBL2003	Nuclear receptor	0.0972399887602	66 / 0
Retinoid X receptor alpha	RXRA	P19793	CHEMBL2061	Nuclear receptor	0.0972399887602	216 / 0
MAP kinase ERK2	MAPK1	P28482	CHEMBL4040	Kinase	0.0972399887602	7 / 0
Purinergic receptor P2Y1	P2RY1	P47900	CHEMBL4315	Family A G protein-coupled receptor	0.0972399887602	1 / 0
Monocarboxylate transporter 4	SLC16A3	O15427	CHEMBL2073663	Electrochemical transporter	0.0972399887602	7 / 0
Cholecystokinin B receptor (by homology)	CCKBR	P32239	CHEMBL298	Family A G protein-coupled receptor	0.0972399887602	116 / 0
G-protein coupled bile acid receptor 1	GPBAR1	Q8TDU6	CHEMBL5409	Family A G protein-coupled receptor	0.0972399887602	4 / 0
PI3-kinase p110-delta subunit	PIK3CD	O00329	CHEMBL3130	Enzyme	0.0972399887602	11 / 10
PI3-kinase p110-gamma subunit	PIK3CG	P48736	CHEMBL3267	Enzyme	0.0972399887602	6 / 13

Target	Common name	Uniprot ID	ChEMBL ID	Target Class	Probability*	Known actives (3D/2D)
Retinoid X receptor beta	RXRB	P28702	CHEMBL1870	Nuclear receptor	0.0972399887602	67 / 0
Retinoid X receptor gamma	RXRG	P48443	CHEMBL2004	Nuclear receptor	0.0972399887602	101 / 0
Receptor-type tyrosine-protein phosphatase F (LAR)	PTPRF	P10586	CHEMBL3521	Membrane receptor	0.0972399887602	21 / 0
Prostanoid EP3 receptor	PTGER3	P43115	CHEMBL3710	Family A G protein-coupled receptor	0.0972399887602	49 / 0
G-protein coupled receptor 35	GPR35	Q9HC97	CHEMBL1293267	Family A G protein-coupled receptor	0.0972399887602	38 / 0
