# Supplementary material for: Identification of AS1842856 as a novel small‐molecule GSK3α/β inhibitor against Tauopathy by accelerating GSK3α/β exocytosis
Source: Aging Cell. 2024 Sep 17;24(1):e14336. doi: 10.1111/acel.14336 (PMC11709109; doi:10.1111/acel.14336)
Supplement: Supplementary file 2 — Tables S2–S3 [file ACEL-24-e14336-s003.docx]

**Supplementary Table 2-3**

Supplementary Table 2: Antibody information.

| Antibody | Company | Catalog # | Application/Dilution |
| --- | --- | --- | --- |
| GSK3α/β | Immunoway | YT2081 | WB (1:1000); IF (1:200) |
| p-Tau (Ser404) | Immunoway | YP0264 | WB (1:1000) |
| p-Tau (Ser396) | CST | 9632S | WB (1:1000); IF (1:200) |
| p-Tau (Thr231) | Thermo | 44-746G | WB (1:1000) |
| p-Tau (Thr205) | Thermo | 44-738G | WB (1:1000); IF (1:300) |
| p-Tau (Ser202) | CST | 39357S | WB (1:1000) |
| p-Tau (Ser199) | CST | 29957S | WB (1:1000) |
| p-Tau (Thr181) | proteintech | 28866-1-AP | WB (1:1000) |
| Tau5 | Santa Cruz | sc-58860 | WB (1:500) |
| Flag | ABMART Inc | M20008 | WB (1:5000) |
| FOXO1 | CST | 2880S | WB (1:1000) |
| p-FOXO1 (Ser256) | Bioss | bs-3142R | WB (1:500) |
| PP2A | CST | 2038S | WB (1:1000) |
| CDK5 | Immunoway | YT0835 | WB (1:1000) |
| p-CDK5 | Immunoway | YP0380 | WB (1:1000) |
| Rab5 | CST | 46449S | WB (1:1000); IF (1:200) |
| NMDAR2A | proteintech | 19953-1-AP | WB (1:500) |
| Alix | CST | 2171 | WB (1:1000); IF (1:50) |
| MAP2 | Santa Cruz | sc-74421 | WB (1:500); IF (1:100) |
| PSD95 | Abcam | ab18258 | WB (1:1000) |
| SYP | proteintech | 17785-1-AP | WB (1:1000) |
| NeuN | Abcam | ab104224 | WB (1:2000); IF (1:500) |
| NeuN | Abcam | ab177487 | IF (1:500) |
| TNF-α | Abcam | ab183218 | WB (1:1000) |
| IL-17 | Abcam | ab79056 | WB (1:1000) |
| IL-1β | CST | 12242 | WB (1:1000) |
| IL-6 | proteintech | 21865-1-AP | WB (1:500) |
| p300 | CST | 86377 | WB (1:1000) |
| AKT | CST | 4685 | WB (1:1000) |
| p-AKT | CST | 4060 | WB (1:1000) |
| β-catenin | ABclonal | A0316 | WB (1:1000) |
| β-actin | ABclonal | AC004 | WB (1:5000) |
| ATP1A1 | proteintech | 14418-1-AP | IF (1:200) |

Supplementary Table 3: The information on compounds.

| Compound | Company | Catalog # | Working concentrations |
| --- | --- | --- | --- |
| AS1842856 | MCE | HY-100596 | 0.5-8 μM |
| MG132 | MCE | HY-13259 | 30 nM |
| CQ | MCE | HY-17589A | 10 μM |
| CCCP | MCE | HY-100941 | 10 μM |
| PMSF | MCE | HY-B0496 | 100 μM |
| Dynasore | MCE | HY-15304 | 10 μM |
| Vacuolin-1 | GLPBIO | GC10325 | 10 μM |
| Marimastat | GLPBIO | GC14099 | 10 μM |
| OA | GLPBIO | GC16958 | 30 nM |
| Endosidin 2 | TOPSCIENCE | T37020 | 0.2 mM |
| GW4869 | Selleck | S7609 | 5 μM |
| SB216763 | MCE | HY-12012 | 0.5 μM |
| AR-A014418 | MCE | HY-10512 | 0.5 μM |
