# Supplementary material for: Identification of AS1842856 as a novel small‐molecule GSK3α/β inhibitor against Tauopathy by accelerating GSK3α/β exocytosis
Source: Aging Cell. 2024 Sep 17;24(1):e14336. doi: 10.1111/acel.14336 (PMC11709109; doi:10.1111/acel.14336)
Supplement: Supplementary file 3 — Appendix S1 [file ACEL-24-e14336-s002.docx]

**Supporting Information**

**Methods**

**Open field test**

The open field test was conducted in a 40 × 40 × 40 cm box. The experimental environment was kept quiet, and the mice moved freely in the box for 5 min. The trajectory and status of the mice were recorded using Smart 3.0 software.

**Vesicle enrichment in cells lysates**

N2a cells were equally inoculated in three 10-cm dishes and divided into IgG, Vehicle and AS groups, and the serum-free medium was replaced when the growth was up to 80% of the culture area for 4 h. The AS group was treated with the addition of AS (0.5 mM) for 6 h, and solvent control was added to the IgG and Vehicle groups. Collection of each group of cells. After that, the three groups of cells were homogenized in 1 mL of 10 mM N-2-hydroxyethylpiperazine-N-ethane-sulphonicacid (HEPES)-sucrose buffer (10 mM HEPES [pH 7.4], 320 mM sucrose, 5 mM MgSO4, 1 mM Ethylene Diamine Tetraacetie Acid (EDTA), and protease inhibitors). 100 uL of each sample was transferred for input. Meanwhile, an appropriate 3 uL per sample amount of mouse anti-Alix (Cell Signaling Technology, 2171) was mixed with pre-cooled magnetic beads on ice and further incubated for 1 h. The Vehicle and AS groups mixtures were then spun overnight with the Alix antibody-coupled magnetic beads at 4 ℃ and the IgG groups mixtures were spun overnight with an equal amount of magnetic beads conjugated mouse IgG antibody (Cell Signaling Technology, 5873S) at 4 ℃. The samples were washed 5 times with 1 mL of HEPES-sucrose buffer on a magnetic rack. Finally, samples were eluted with sample buffer and heat denatured at 100 °C for 5 min for subsequent Western blot detection.

**Supplemental Results**


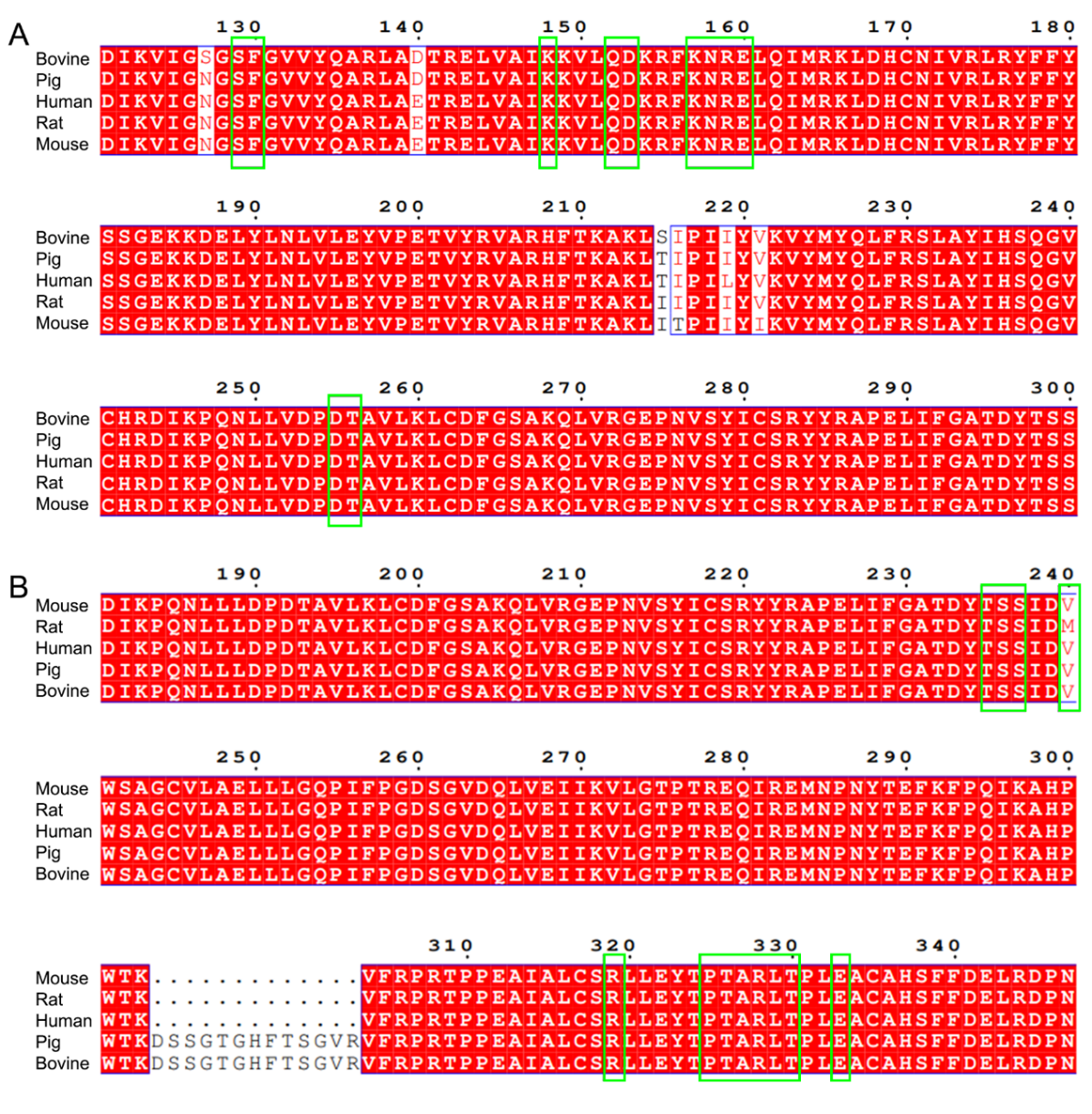


**Figure S1.** Homology of GSK3α/β in different species. (A-B) Homology of GSK3α (A) and GSK3β (B) in mouse, rat, human, pig and bovine. The green boxes labeled the amino acids that could bind to AS.

**
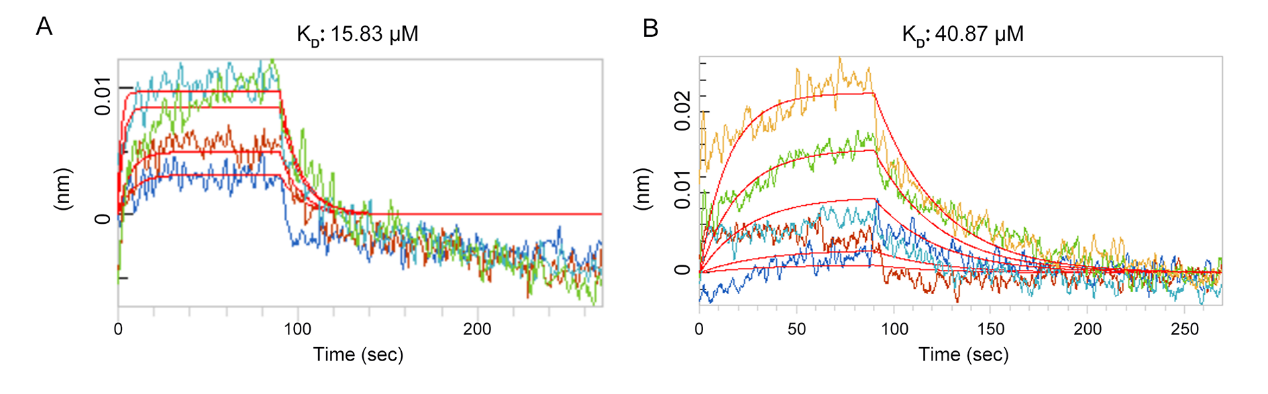
 Figure S2.** Binding affinity of GSK3α and GSK3β to AS.(A) The binding affinity of GSK3α to AS, and the K_D_ values is 15.83 μM. (B) The binding affinity of GSK3β to AS, and the K_D_ values is 40.87 μM.


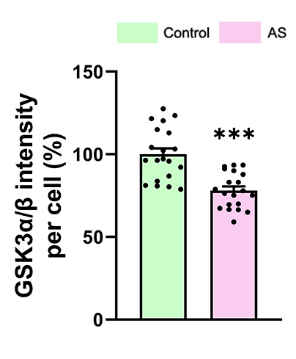


**Figure S3.** The statistical graph of Figure 1M. n = 20; ****P* < 0.001.


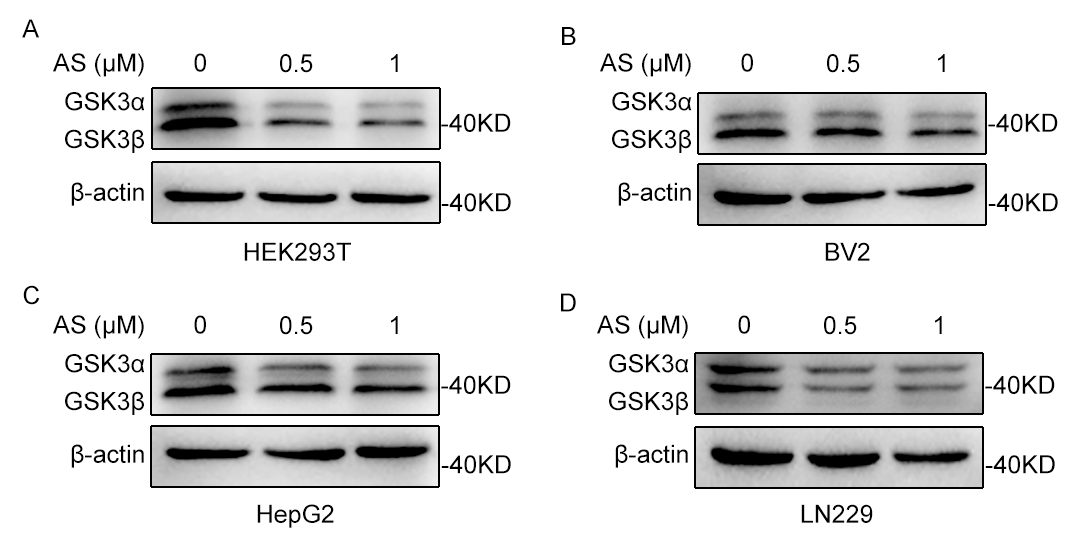


**Figure S4.** Consequences of AS treatment on GSK3α/β expression levels in multiple cells. Western blot depicting changes in the levels of GSK3α/β in HEK293T cells (A), BV2 cells (B), HepG2 cells (C) and LN229 cells (D) following AS treatment for 12 h. n = 3.


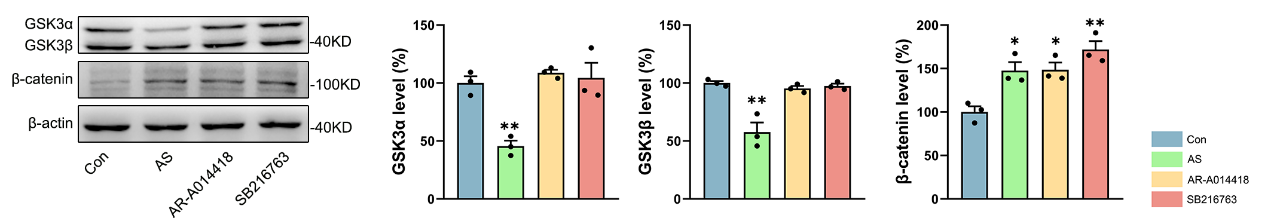


**Figure S5.** In contrast to AR-A014418 and SB216763, only AS was found to reduce GSK3α/β levels. N2a cells were treated with AS (0.5 μM), AR-A014418 (0.5 μM) or SB216763 (0.5 μM) for 6 h. GSK3α/β and β-catenin levels were detected using Western blot. n = 3; **P* < 0.05, ***P* < 0.01.


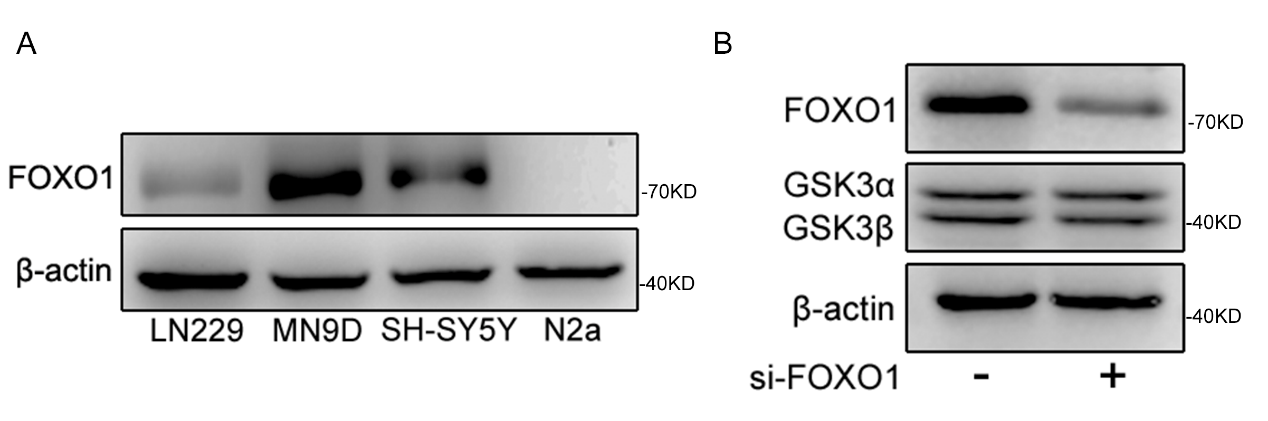


**Figure S6.** FOXO1 knockdown did not change the expression level of GSK3α/β. (A) Western blots showed the expression levels of FOXO1 in LN229, MN9D, SH-SY5Y and N2a cells. (B) The expression of GSK3α/β remained unaltered after FOXO1 knockdown in MN9D cells. n = 3.


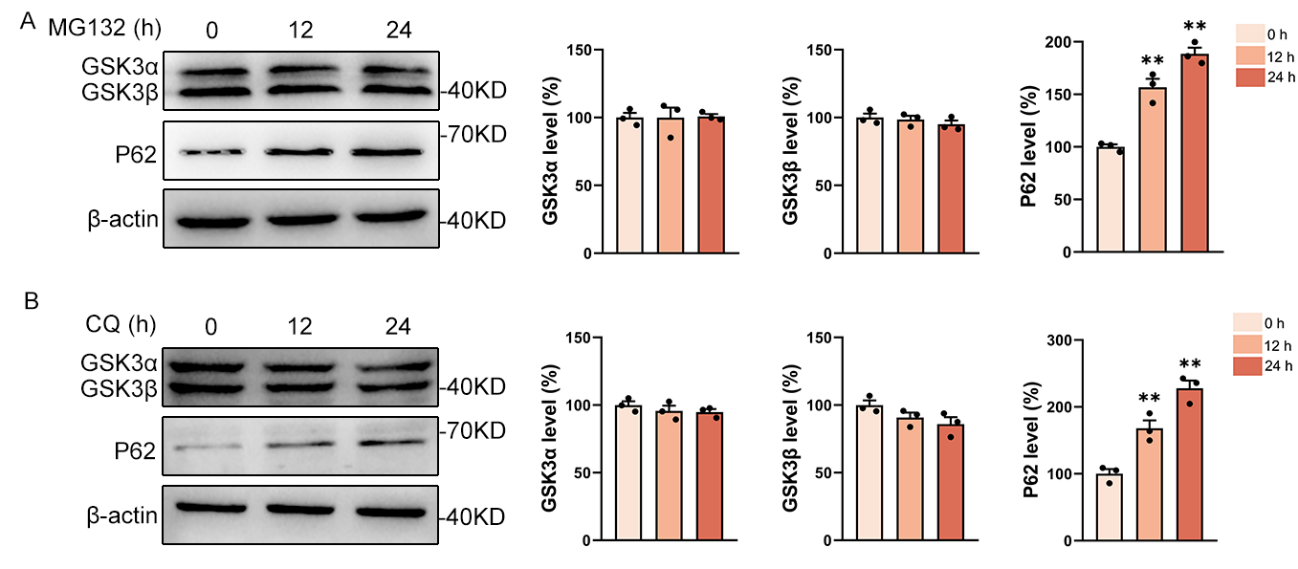


**Figure S7.** MG132 and CQ have no significant effects on GSK3α/β levels in N2a cells. MG132 (A, 30 nM) and CQ (B, 10 μM) were used to treat N2a cells for 12 h and 24 h. Western blot was used to detect levels of GSK3α/β and P62. n = 3; ***P* < 0.01.


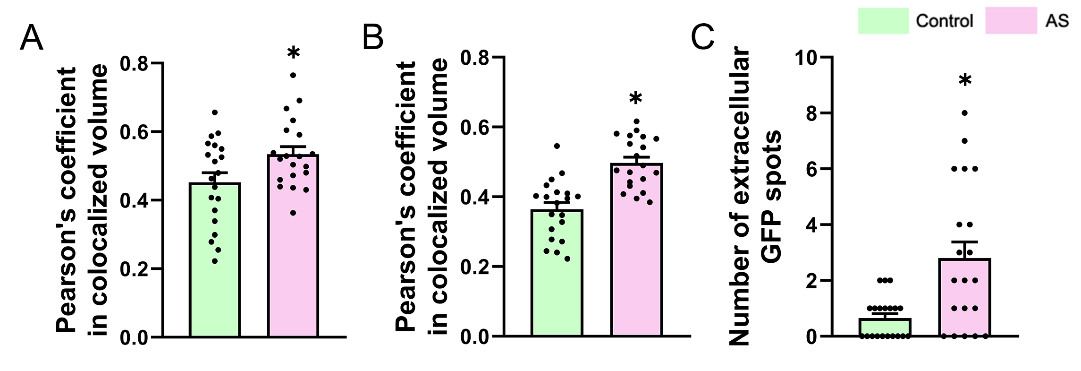


**Figure S8.** The statistical graphs of Figure 2E (A), Figure 2F (B) and Figure 2G (C). n = 20; **P* < 0.05.


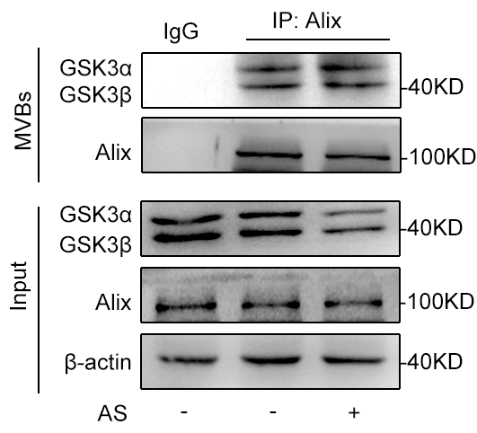


**Figure S9.** AS treatment increases the content of GSK3α/β in the Alix-positive vesicles from N2a cells.


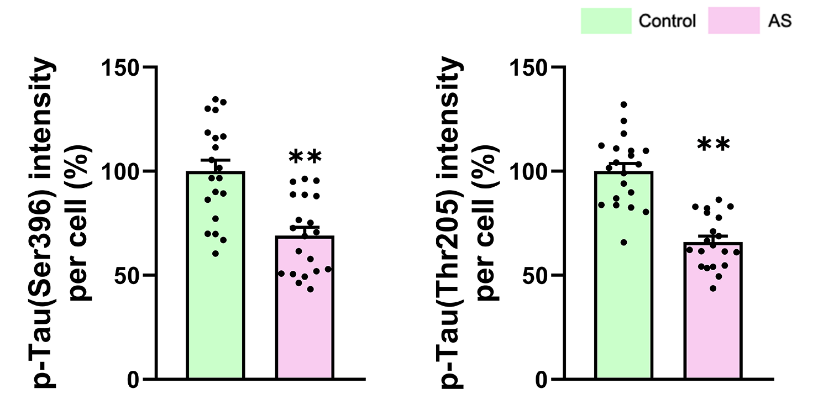


**Figure S10.** The statistical graphs of Figure 3E. n = 20; ***P* < 0.01.


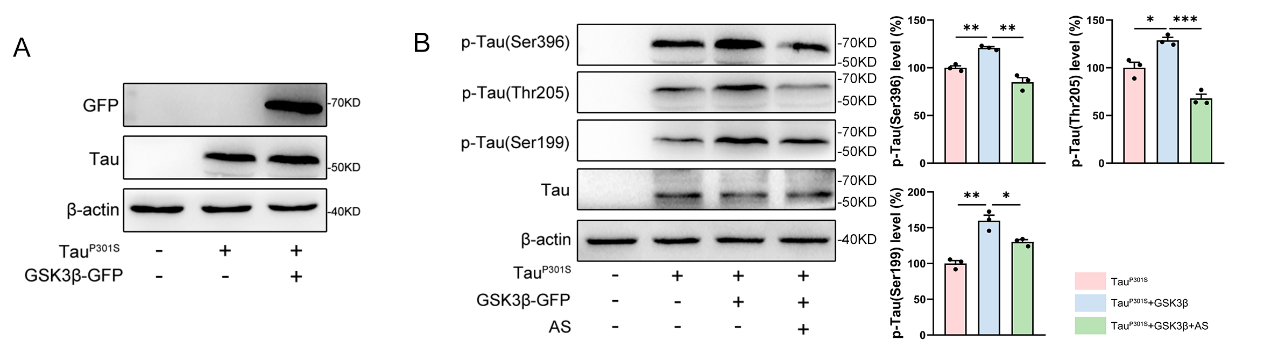


**Figure S11.** AS treatment rescues GSK3β overexpression-induced Tau hyperphosphorylation. (A) Western blot evaluated the expressions of GFP and Tau after overexpression of Tau^P301S^ and GSK3β-GFP in HEK293T cells. (B) AS (0.5μM) treatment for 6 h inhibited Tau hyperphosphorylation at Ser396, Thr205 and Ser199 in HEK293T cells overexpressing Tau^P301S^ and GSK3β-GFP mutant. n = 3; **P* < 0.05, ***P* < 0.01, ****P* < 0.001.

**
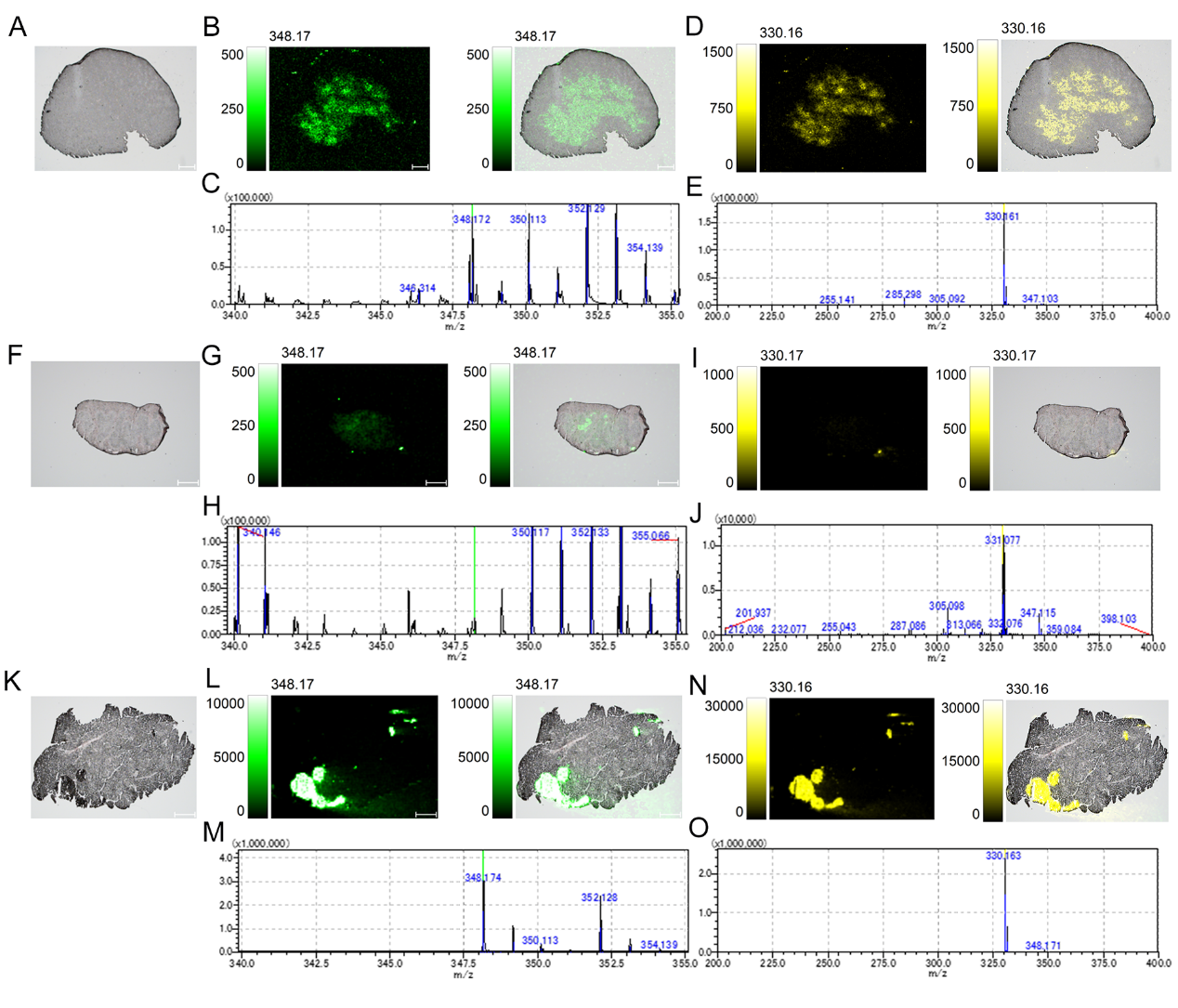
 Figure S12.** MS and MS/MS imaging-based visual mapping profiles of AS1842586. (A-D) MS and MS/MS imaging visualized the spatial distributions of AS in the liver. (F-H) MS and MS/MS imaging visualized the spatial distributions of AS in the spleen. (K-M) MS and MS/MS imaging visualized the spatial distributions of AS in the pancreas. All ion images were normalized to the CHCA matrix signal (pixel size, 40 μm). MALDI-MSI single pixel mass spectra from the liver (C-E), spleen (H-J), and pancreas (M-O) tissues with AS.


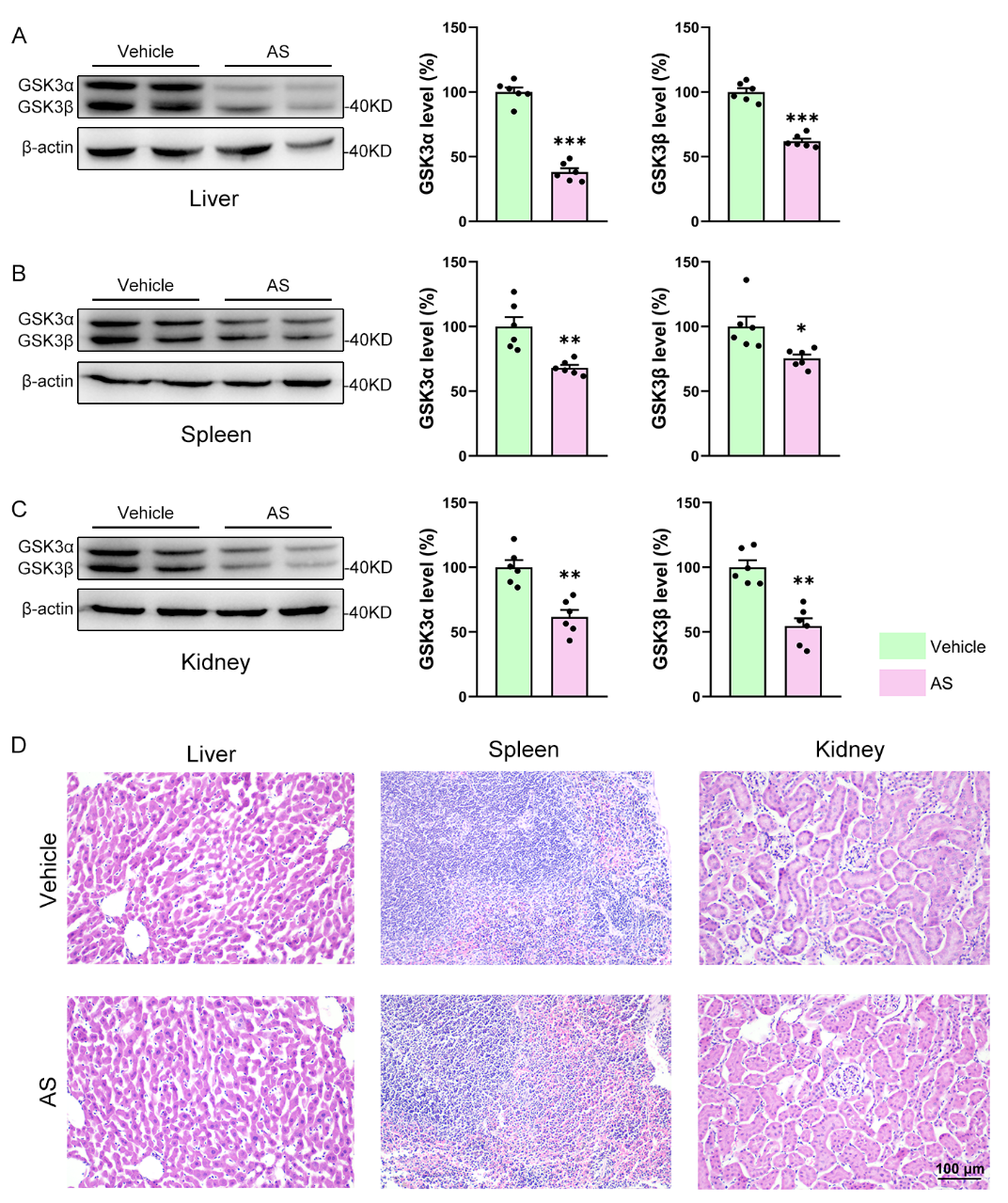


**Figure S13.** AS reduces GSK3α/β levels in the liver, spleen and kidney tissues without causing histopathologic changes. Western blot was used to evaluate the expressions of GSK3α/β in the liver (A), spleen (B) and kidney (C) of P301S mice after treatment with AS for 8 weeks. n = 6; **P* < 0.05, ***P* < 0.01, ****P* < 0.001. (D) H&E staining was used to analyze the morphological changes of the liver, spleen and kidney.


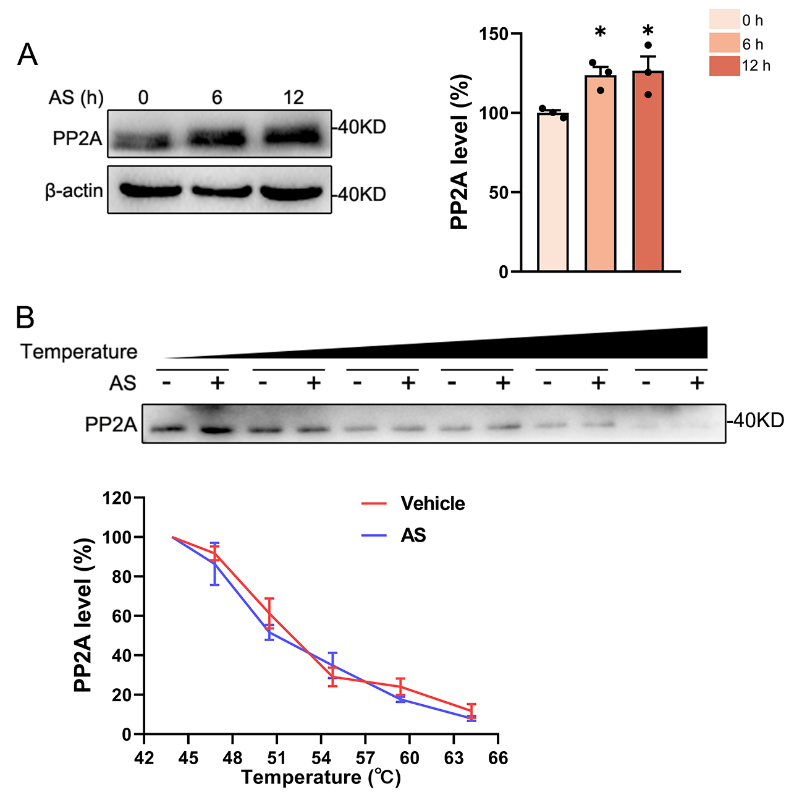


**Figure S14.** AS indirectly increases the expression level of PP2A in N2a cells. (A) AS (0.5μM) was used to treat N2a cells for 6 h and 12 h. Western blot was used to detect PP2A levels. n = 3; **P* < 0.05. (B) Western blot quantified the level of PP2A after AS treatment in CETSA, the melting curves of PP2A were affected by temperature. n = 3.


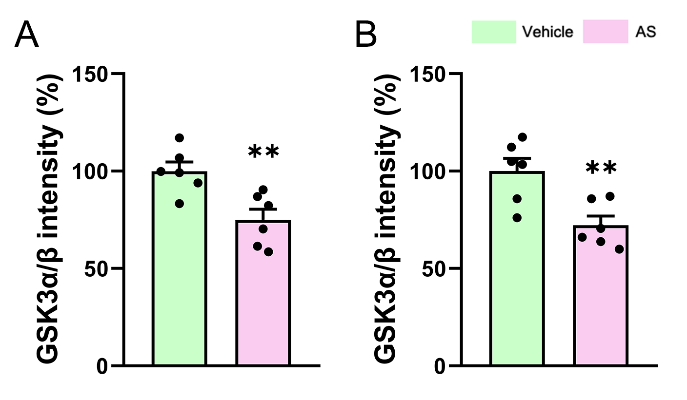


**Figure S15.** The statistical graphs of Figure 4I (A) and Figure 4J (B). n = 6; ***P* < 0.01.


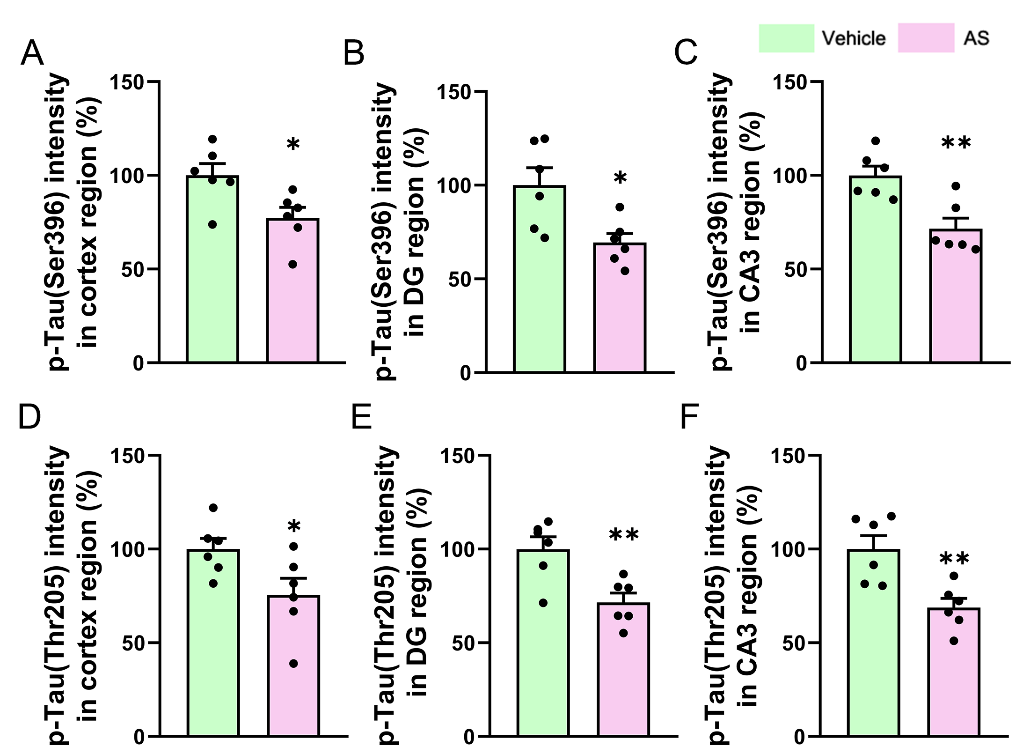


**Figure S16.** The statistical graphs of Figure 5B. (A-C) The graphs show the statistical data of p-Tau(Ser396) intensity in the cortex (A), DG (B) and CA3 (C) region. (D-F) The graphs show the statistical data of p-Tau(Thr205) intensity in the cortex (D), DG (E) and CA3 (F) region. n = 6; **P* < 0.05, ***P* < 0.01.


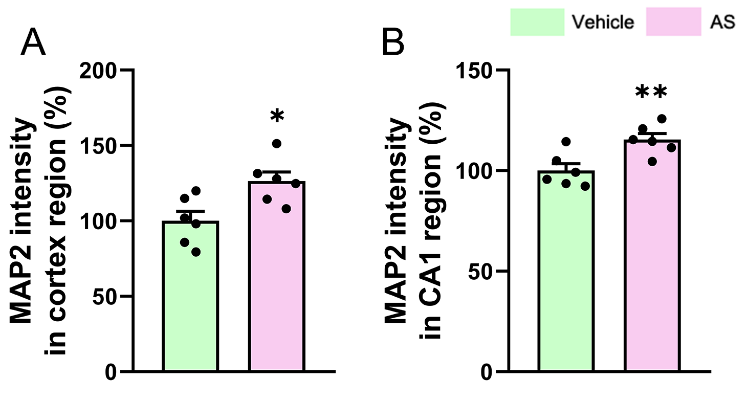


**Figure S17.** The statistical graphs of Figure 6B (A) and Figure 6C (B). n = 6; **P* < 0.05, ***P* < 0.01.


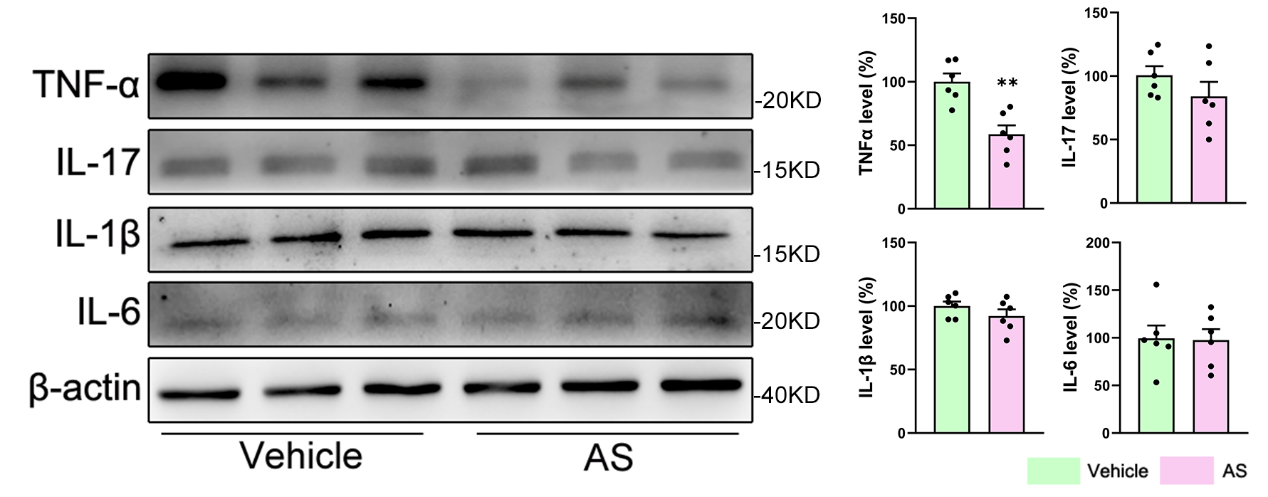


**Figure S18.** AS reduces neuroinflammation in P301S transgenic mouse brains. Western blot evaluated the expression levels of TNF-α, IL-17, IL-1β and IL-6 in P301S transgenic mouse hippocampus. n = 6; ***P* < 0.01.


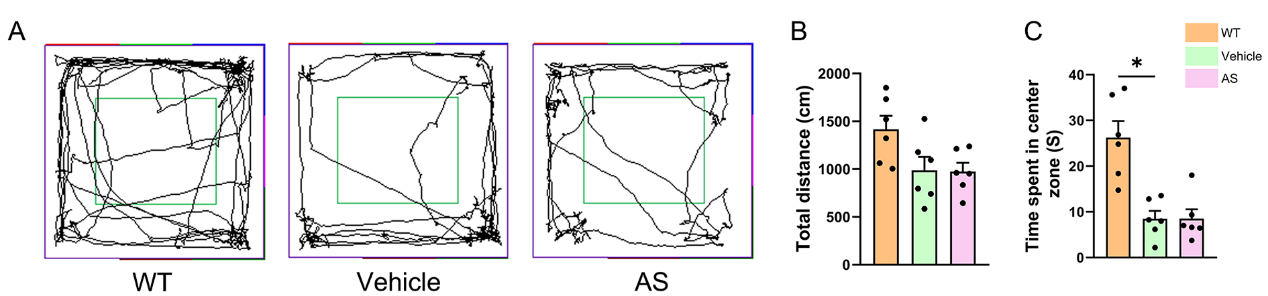


**Figure S19.** AS treatment did not improve anxiety-like behaviors in P301S transgenic mice. (A) The typical traveling traces of the mice during 5 min of open field exploration. (B) The total distances of three indicated groups of mice during 5 min of open field exploration. (C) Statistical graph of three indicated groups of mice for duration in the center zone. n = 6; **P* < 0.05.
